# Preventable medication harm across health care settings: a systematic review and meta-analysis

**DOI:** 10.1186/s12916-020-01774-9

**Published:** 2020-11-06

**Authors:** Alexander Hodkinson, Natasha Tyler, Darren M. Ashcroft, Richard N. Keers, Kanza Khan, Denham Phipps, Aseel Abuzour, Peter Bower, Anthony Avery, Stephen Campbell, Maria Panagioti

**Affiliations:** 1 National Institute for Health Research School for Primary Care Research, Centre for Primary Care and Health Services Research, Division of Population Health, Health Services Research and Primary Care, School of Health Sciences, Faculty of Biology, Medicine and Health, University of Manchester, Manchester Academic Health Science Centre, Williamson Building, Oxford Road, Manchester, M13 9PL UK; 2 National Institute for HealthResearch Greater Manchester Patient Safety Translational Research Centre, School of Health Sciences, University of Manchester, Manchester, M13 9PL UK; 3 Centre for Pharmacoepidemiology and Drug Safety, Division of Pharmacy and Optometry, University of Manchester, Manchester, UK; 4 Pharmacy Department, Greater Manchester Mental Health NHS Foundation Trust, University of Manchester, Manchester, M25 3BL UK; 5 Division of Primary Care, School of Medicine, University of Nottingham, Nottingham, NG7 2RD UK

**Keywords:** Patient safety, Preventable medication harm, Prevalence, Meta-analysis, Medication error

## Abstract

**Background:**

Mitigating or reducing the risk of medication harm is a global policy priority. But evidence reflecting preventable medication harm in medical care and the factors that derive this harm remain unknown. Therefore, we aimed to quantify the prevalence, severity and type of preventable medication harm across medical care settings.

**Methods:**

We performed a systematic review and meta-analysis of observational studies to compare the prevalence of preventable medication harm. Searches were carried out in Medline, Cochrane library, CINAHL, Embase and PsycINFO from 2000 to 27 January 2020. Data extraction and critical appraisal was undertaken by two independent reviewers. Random-effects meta-analysis was employed followed by univariable and multivariable meta-regression. Heterogeneity was quantified using the *I*^2^ statistic, and publication bias was evaluated. PROSPERO: CRD42020164156.

**Results:**

Of the 7780 articles, 81 studies involving 285,687 patients were included. The pooled prevalence for preventable medication harm was 3% (95% confidence interval (CI) 2 to 4%, *I*^2^ = 99%) and for overall medication harm was 9% (95% CI 7 to 11%, *I*^2^ = 99.5%) of all patient incidence records. The highest rates of preventable medication harm were seen in elderly patient care settings (11%, 95% 7 to 15%, *n* = 7), intensive care (7%, 4 to 12%, *n* = 6), highly specialised or surgical care (6%, 3 to 11%, *n* = 13) and emergency medicine (5%, 2 to 12%, *n* = 12). The proportion of mild preventable medication harm was 39% (28 to 51%, *n* = 20, *I*^2^ = 96.4%), moderate preventable harm 40% (31 to 49%, *n* = 22, *I*^2^ = 93.6%) and clinically severe or life-threatening preventable harm 26% (15 to 37%, *n* = 28, *I*^2^ = 97%). The source of the highest prevalence rates of preventable harm were at the prescribing (58%, 42 to 73%, *n* = 9, *I*^2^ = 94%) and monitoring (47%, 21 to 73%, *n* = 8, *I*^2^ = 99%) stages of medication use. Preventable harm was greatest in medicines affecting the ‘central nervous system’ and ‘cardiovascular system’.

**Conclusions:**

This is the largest meta-analysis to assess preventable medication harm. We conclude that around one in 30 patients are exposed to preventable medication harm in medical care, and more than a quarter of this harm is considered severe or life-threatening. Our results support the World Health Organisation’s push for the detection and mitigation of medication-related harm as being a top priority, whilst highlighting other key potential targets for remedial intervention that should be a priority focus for future research.

## Background

The Institute of Medicine report ‘*To Err is Human: Building a Safer health System*’ helped generate the patient safety movement by reporting that up to 98,000 deaths were due to medication and at least some of them could have been preventable [1–3]. On March 2017, the World Health Organisation (WHO) launched a global initiative to develop approaches to reduce severe, preventable medication harm in all countries by 50% over the next 5 years [4]. Similarly, in May 2018, the Institute for Healthcare Improvement of the USA launched a multi-organisation initiative to create a national action plan for the prevention of harm in healthcare [5].

In the USA alone, medication errors are the third most common cause of death behind heart disease and cancer [6] accounting for injuries to approximately 1.3 million people annually [7, 8]. Similarly, in the UK, it was estimated that there are 237 million medication errors in England over a year period [9, 10], and preventable adverse drug reactions were estimated to cost the National Health Service (NHS) £98.5 million per annum, consume 181,626 bed days, cause 712 deaths and contribute to 1708 deaths during initial hospitalisation.

It is important to underline that not all harms caused by medication errors are preventable. However, distinction between preventable and non-preventable harm is becoming increasingly important, because it provides an indication of where best to invest limited resources for healthcare improvement in order to have the greatest benefit. From a quality improvement perspective, measuring harm, particularly preventable harm, alongside medication error [11] is critical. This would help ensure efforts are more patient-centred; can target the system rather than the individual, with the aim of enhancing clinical outcomes; reduce punitive concerns associated with the reporting methodology; allow for analysis of unintended results and encourage learning from events to continually improve the processes for detecting preventable harm [12]. Practitioners should also supplement preventable medication harm assessment with the measurement of certain contributory factors such as the severity of harm, the source at which the harm occurred (i.e. prescribing, transcribing, dispensing, administering or monitoring) and classification of harm according to the ‘five rights’ (patient, drug, dose, route, and time). Each of these factors could significantly inform quality improvement efforts [13].

Due to the ongoing global challenges in this area, it is important that healthcare providers, researchers and policy makers have a better understanding about the current prevalence rates and nature of preventable medication harm. Therefore, we undertook a systematic review and meta-analysis to inform the identification of targets for improvement efforts on estimating the prevalence of preventable medication harm across medical care settings including hospitals, primary care and various specialties. We also examined the clinical severity of preventable medication harm, and the impact of the medication use, stage and high-risk medication groups by building on what is already known from previous research efforts [14–16].

## Methods

This systematic review was conducted and reported in accordance with the Reporting Checklist for Meta-analyses of Observational Studies (MOOSE). The completed MOOSE checklist is available in Additional file 1: Table S1. The review protocol is registered in PROSPERO under the review number: CRD42020164156.

### Data sources and searches

We searched the five electronic bibliographic databases from 2000 to 27 January 2020: Medline, Cochrane library, Embase and PsycINFO via Ovid and CINAHL via EBSCO. The searches were supplemented by checking conference abstracts and screening grey literature sources (WHOLIS, Google Scholar, SIGLE). We also identified eligible studies by checking references in existing systematic reviews in the area. The full search strategy is available in the Additional file 2: Table S2.

### Eligibility criteria

We included observational studies (retrospective and prospective cohorts; cross-sectional studies) in any medical care setting (primary, secondary and tertiary care) published from January 2000 onwards in English language. This date was selected, because it coincides with the publication of landmark patient safety reports, the increase of patient safety research in volume and the assessment of preventability [1, 17, 18]. The primary outcome was the prevalence of preventable medication-related harm including [19] adverse drug events (ADEs) or adverse drug reactions (ADRs) whether they were acts of omission or commission, incorrect medication/dose/timing, administration of a medication to a patient with a known allergy, inadequate monitoring or other errors. For inclusion in this study, the study authors clearly defined their inclusion criteria as being about ADEs or ADRs and assessment of preventability. We only included studies that provide amenable data for inclusion in meta-analysis. Studies reporting medication errors or non-adherence and that involved only patients with re-admissions due to recurring medication harm were excluded.

### Study selection and data extraction

The titles and abstracts of all identified citations as well as potentially eligible full texts were screened independently by two reviewers (AH, MP), using pre-defined criteria. Disagreements were settled through consultation with a third team member (RNK). For eligible studies, we used a pilot-tested extraction spreadsheet, to extract descriptive data on study characteristics (e.g. number and age of participants, research design, systems used for data collection, assessment and preventability) and quantitative outcomes (prevalence, severity, medical care setting, the medication group and stage of medication use of preventable medication harm). Two independent researchers (AH and NT) performed the data extraction with disagreements resolved by discussion within the wider team (MP, RNK, DMA).

### Risk of bias assessment

The quality of the studies was evaluated by two independent reviewers (AH and NT) using the Newcastle-Ottawa scale for cross-sectional and cohort studies. This assessed the representativeness of the sample size, response rate, ascertainment of the exposure, control of confounding variables, assessment of preventability and timing and appropriate statistical analysis, which provided a score ranging from 0 (lowest grade) to 10 (highest grade). A higher grade indicated a lower risk of bias. For our analyses, studies scoring 7 or above were considered as low risk, whereas studies scoring below 7 were considered as high risk of bias.

### Data analysis

Prevalence of preventable medication harm was pooled using random-effects models with the DerSimonian-Laird approach [20]. Secondary analysis looked at WHO priority areas including the healthcare system setting (i.e. general hospital/internal medicine; emergency department or ICU; highly specialised or surgical care—which included hospitals with long-term acute care facilities for dealing with certain disease categories such as cardiac, oncology, or orthopaedic problems; care units for paediatric patients, including neonatal intensive care units; and elderly patients, including geriatric patients aged above 64 years), the severity (i.e. mild, moderate, severe/life-threatening), stage of medication (i.e. using the US Pharmacopeia five major steps [21]: prescribing, transcribing and documenting, dispensing, administering and monitoring), patient age distribution (new born (less than 3 years), adolescents (3 to 18 years), adults (19 to 69 years), elder (70 years or above)) based on mean or median and medication group according to the Anatomical Therapeutic Chemical (ATC) Classification System [22].

Unadjusted prevalence, incidence and standard errors for the study-specific estimates were recalculated, based on the information of crude numerators and denominators provided in the individual studies. To keep the effect of studies with extremely small prevalence estimates on the overall estimate to a minimum, the variance of the study-specific prevalence was stabilised with the Freeman-Tukey double arcsine transformation [23]. Sensitivity analyses were done using other transformations, a range of sample sizes and adjustments for between-study variance (*τ*^2^) [24–27].

Univariable and multivariable meta-regressions were performed to investigate possible sources of heterogeneity using the following variables: WHO region (North America, Europe and Asia), drug class, median sample size, age group (adolescent, adults or mixed), medical setting (same as healthcare system setting), assessment method (medical record review or survey/telephone/voluntary report), standard preventability assessment method (i.e. Hallas criteria [28] or Schumock and Thornton scale [29]) and severity classification system, length of study (less than 6 months or more), design (prospective cohorts, retrospect cohorts or cross-sectional) and sensitivity analysis with studies at lower risk of bias.

Heterogeneity was assessed using the *χ*^2^ test on Cochrane’s *Q* statistic and quantified by calculating *I*^2^ [30]. Values of 25%, 50% and 75% for *I*^2^ represent low, medium and high heterogeneity [31]. The presence of publication bias was assessed by inspection of funnel plots, the Egger’s test and the trim-and-fill method. All analyses were done using the ‘meta’ and ‘metafor’ packages of the statistical software R (v.3.6.3) [32]; specifically, the ‘metaprop’ function was used for pooling prevalence rates.

### Patient and public involvement

Six patients who were members of our research advisory panel were involved in the development of our research questions, research protocol and selection of outcomes and they advised on the interpretation and dissemination of results.

## Results

**Fig. 1:**
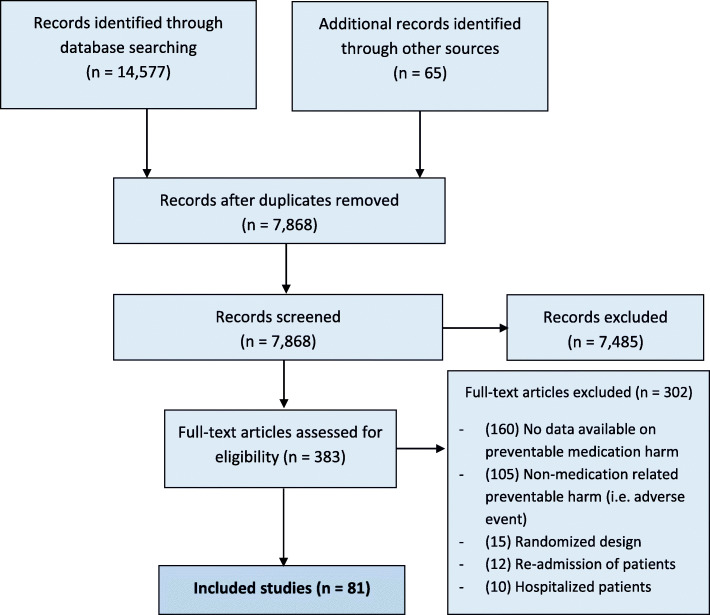
PRISMA flowchart of the included studies

The searches revealed 7868 articles, and after reviewing the full text screening, 81 studies met the inclusion criteria (see PRISMA flow chart in Fig. 1). A list of the eligible studies is included in Additional file 3: Table S3.

### Characteristics of included studies

Twenty-six of the studies (32%) were conducted in Europe, 23 studies (28%) in the USA, and the rest of the studies were mainly carried out in South East Asia, South America or the Middle East. The median sample size across studies was 1234 patients (IQR estimate 2652). The pooled sample size across the 81 eligible studies was 285,687 patients. Of these, 18,243 (6.4%) patients experienced at least one classified medication related harm, and 7075 (2.5%) experienced at least one preventable medication harm. In total, 20,698 medication harm incidents were reported, of which 7589 (37%) were preventable medication harm.

Fifty-eight studies (72%) used prospective cohort designs, 15 (19%) retrospective cohort designs and eight (10%) cross-sectional designs (Additional file 4: Table S4). The most common setting of medical care was general hospitals or internal medicine settings in 29 studies (36%). Fourteen (17%) studies involved patients subject to highly specialised care (seven of which included patients in surgery [33–39] and two involving psychiatric patients [40, 41]), twelve (15%) studies were situated in an emergency department, six (10%) in an ICU and four (5%) studies in primary care. The focus in nine (11%) of the studies was on paediatric care units, with three of these studies being situated in neonatal intensive care units [42–44]. Seven (9%) studies involved elderly patient care units, six of which were specialised care units for geriatric patients aged above 65 years [35, 45–49].

Preventable medication harm was assessed in 54 (67%) studies by use of medical record reviews or observations. In contrast, 27 (33%) studies reported preventable harm by survey, telephone or spontaneous reporting surveillance systems. At least one of the standard methods [28, 29] for assessing the preventability of medication harm involving consensus between two or more trained reviewers (physicians or nurses) was applied in 48 (59%) studies. However, 20 (25%) of the studies used adapted scales where consensus procedures were not well known, and 13 studies (16%) had not defined the method used for assessment of preventability.

### Quality assessment

The median Newcastle-Ottawa score for included studies was 7 (range 6 to 9). Thirty-five studies (43%) scored eight or above and were considered to be at low risk of bias (see full assessment in Additional file 5: Table S5).

### Pooled prevalence rates of medication-related harm

Table 1 shows that the unadjusted pooled prevalence of preventable medication harm was 3% (95% CI 2 to 4%, *I*^2^ = 99%) and the pooled prevalence of all medication harm (inclusive of non-preventable) was 9% (95% CI 7 to 11%, *I*^2^ = 99.5%).

**Table 1:** Proportions of the different types of preventable medication harm and overall medication harm

Outcome		Preventable medication harm			Any medication harm		
	Number of studies	Prevalence rate, % (95% CI) *	*I*^2^, %	Median (IQR)	Prevalence rate, % (95% CI) *	*I*^2^, %	Median (IQR)
Overall prevalence rate	81	3 (2, 4)	99	2.9 (0.02–27.1)	9 (7, 11)	99.5	9.8 (0.24–65.4)
*Healthcare system:*							
- General hospital or internal medicine	29	2 (1, 2)	98.4	1.6 (1.2–2.2)	5 (4, 7)	99.0	5.1 (3.2–10.9)
- Emergency department	12	5 (2, 12)	99.7	5.2 (2.1–12)	9.3 (4, 21)	99.8	6.2 (6.0–14)
- ICU	6	7 (4, 12)	91.1	6.8 (3.9–11.6)	14 (8, 23)	95.6	16.4 (9.5–20.2)
- Highly specialised care or surgical	13	6 (3, 11)	98.7	5.4 (3.0–9.7)	18 (11, 26)	99.3	22.9 (17.8–26.2)
- Paediatric	9	1 (0, 2)	94.9	1.1 (0.1–2.1)	5 (2, 10)	99.2	5 (2.4–14.3)
- Elderly	7	11 (7, 15)	95.9	10.7 (7.2–15.4)	23 (13, 37)	99.1	24.8 (16–31.3)
- Primary care	4	1 (1, 2)	84.9	1.2 (1–1.9)	10 (6, 16)	99.0	9.9 (8–13.9)
- Psychiatric	1	1 (1, 2)	NA	NA	10 (9, 12)	NA	NA
*Severity of preventable harm:*							
- Mild	NA	39 (28, 51), *n* = 20	96.4	40 (21–49)	50 (44, 56), *n* = 47	98	46.9 (35.9–63.1)
- Moderate	NA	40 (31, 49), *n* = 22	93.6	36.5 (28.1–54.4)	41 (36, 47), *n* = 54	98	37.3 (24.7–53.3)
- Severe/life-threatening	NA	26 (15, 37), *n* = 28	97	19.5 (8–40)	14 (10, 19), *n* = 55	98	10 (6.4–18.6)
*Stages of medication use*^*β*^*:*							
1. Prescribing	9	58 (42, 73), *n* = 9	94	55.8 (35.4–77.3)	64 (34, 84), *n* = 18	98.5	52 (36–79)
2. Transcribing/documenting	8	3 (0, 9), *n* = 8	78	0.9 (0–6.3)	6 (1, 14), *n* = 17	89	2 (0.5–9)
3. Dispensing	6	2 (0, 6), *n* = 6	47	3.8 (0.9–4.1)	8 (2, 24), *n* = 15	69%	5.6 (1.3–8)
4. Administering	15	21 (11, 33), *n* = 15	93	20.8 (8.8–31.1)	25 (12, 40), *n* = 32	96.8	19 (7.6–30)
5. Monitoring	8	47 (21, 73), *n* = 8	99	54.9 (25.3–76)	54 (16, 82), *n* = 15	100	35 (15–70)

*Freeman-*Tukey double* arcsine transformation was used for the prevalence rate calculations

^β^Stages of medication use follow the using the USA’s Pharmacopeia 5 major steps

**Fig. 2:**
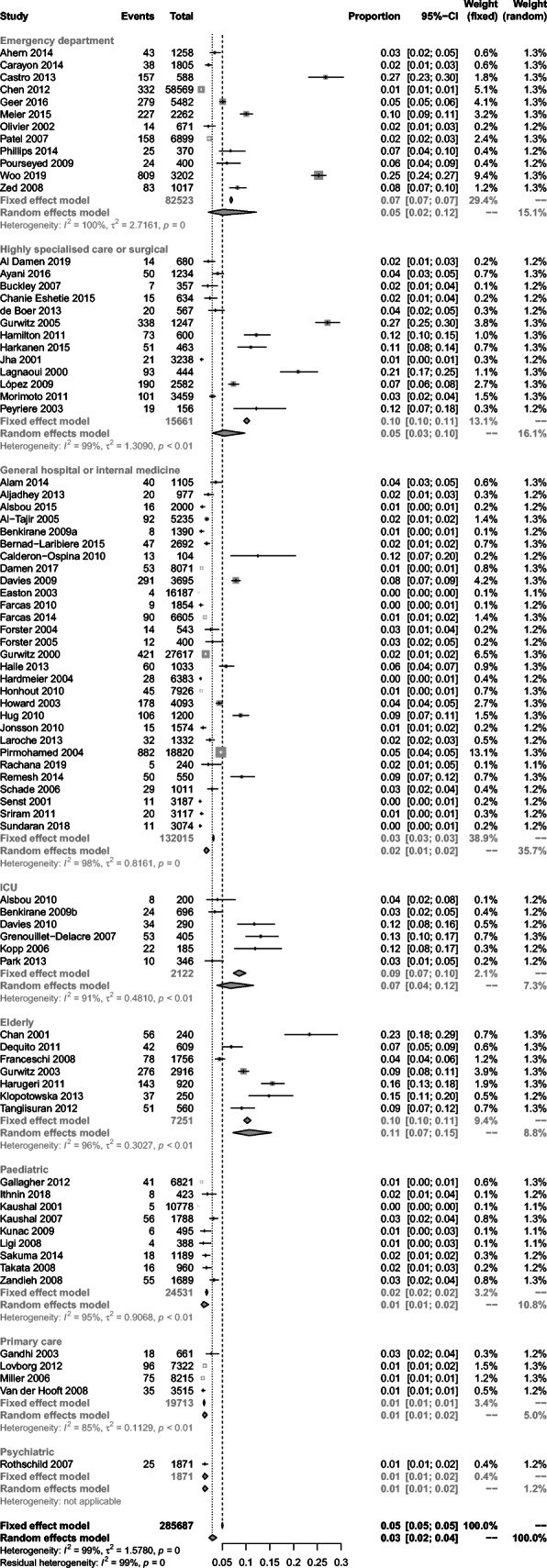
Prevelance rates for preventable medication harm by healthcare system

**Fig. 3:**
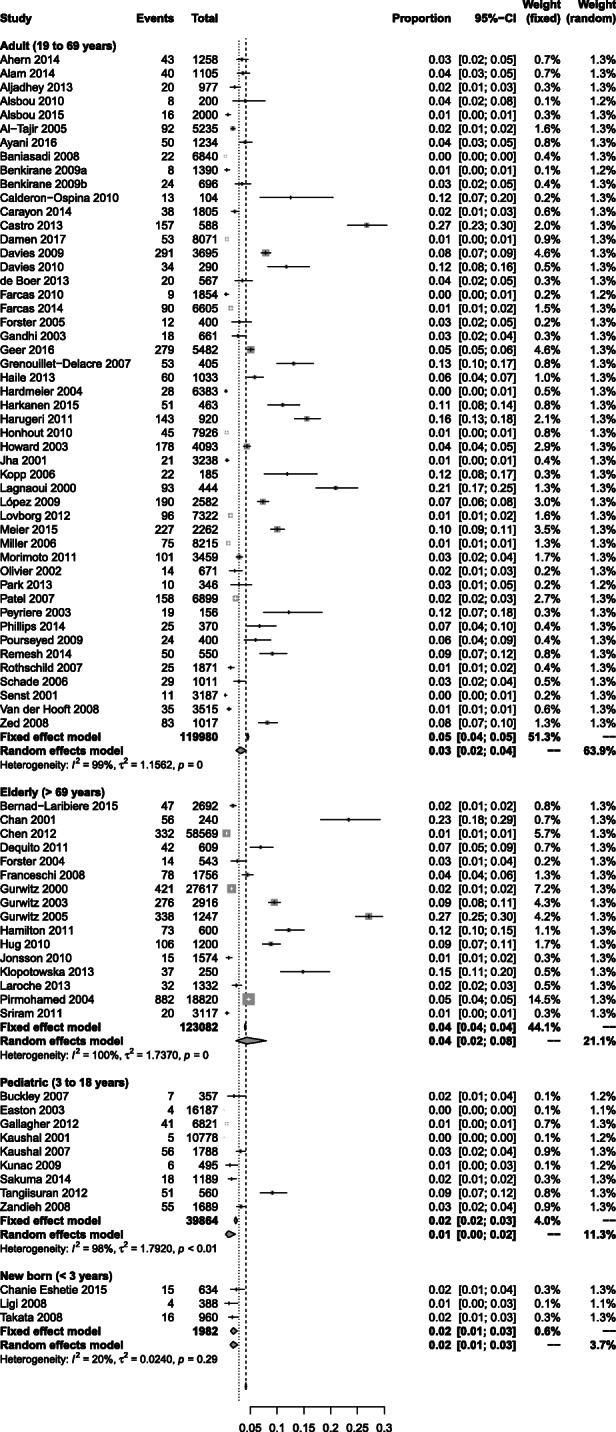
Prevelance rates for preventable medication harm by age group

The highest prevalence rates for preventable medication harm were observed in elderly patient care units (11%, 95% CI 7 to 15%, *n* = 7)), and patients in ICUs (7%, 4 to 12%, *n* = 6)), highly specialised or surgical care (6%, 3 to 11%, *n* = 13)) and emergency departments (5%, 2 to 12%, *n* = 12)) (Fig. 2). In contrast, the lowest prevalence rates were observed in paediatric care units (1%, 0 to 2%, *n* = 9), primary care (1%, 1 to 2%, *n* = 4) and general hospitals or internal medicine settings (2%, 1 to 2%, *n* = 29). Prevalence rates by patient age group are provided in Fig. 3. These results re-enforce earlier findings, showing greater preventable medication harms in elderly patients (4%, 2 to 8%, *n* = 16) and the lowest rate of preventable medication harm in paediatric patients (1%, 0 to 2%, *n* = 9).

### Clinical severity, stage of medication use and medication group

As shown in Table 1, the proportions of clinical severity for mild preventable medication harm was 39% (95% CI 28 to 51%, *n* = 20, *I*^2^ = 96.4%), moderate preventable harm was 40% (31 to 49%, *n* = 22, *I*^2^ = 93.6%) and severe or potentially life-threatening preventable harm was 26% (15 to 37%, *n* = 28, *I*^2^ = 97%).

The highest rates of preventable medication harm were sourced at the prescribing (58%, 95% 42 to 73%, *n* = 9, *I*^2^ = 94%) and monitoring (47%, 95% 21 to 73%, *n* = 8, *I*^2^ = 99%) stages. The stages with the lowest rates of preventable medication harm were sourced at dispensing (2%, 95% 0 to 6%, *n* = 6, *I*^2^ = 47%) and transcribing/documenting (3%, 95% 0 to 9%, *n* = 8, *I*^2^ = 78%) stages.

Preventable harm was greatest in medicines affecting the ‘central nervous system’ (ATC code N) (21%, 95% 6 to 43%, *n* = 6) (see Table 2). However, prevalence rates were also high in the ATC medication groups for ‘cardiovascular’ (code C) (16%, 95% 11 to 23%, *n* = 14), ‘hypnotics and sedatives’ (code N05C) (16%, 95% 11 to 21%, *n* = 5), ‘anti-inflammatory and antirheumatic products’ (code M01) (15%, 95% 7 to 26%, *n* = 5) and ‘antibiotics and antibacterial for systemic use’ (code J01) (12%, 95% 7 to 18%, *n* = 12). The prevalence rate was below 5% for patients receiving ‘respiratory system-related drugs’ (code R), drugs for patients with ‘functional gastrointestinal disorders’ (code A03), patients taking ‘corticosteroids for systemic use’ (code H02) and ‘antiepileptics’ (code N03). The top five medication groups associated with preventable medication harm in each of the 20 studies is provided in Additional file 6: Table. S6.

**Table 2:** Proportions of preventable medication harm by medication class

Drug class	ATC code	***N*** studies	Prevalence, %	95% CI	***τ***^**2**^	*I*^2^ (%)
Analgesics	N02^¥^	4	7	0.5, 19	0.0229	84.00
Antibiotics and antibacterial	J01^¥^	12	12	7, 18	0.0165	87.80
Antithrombotic agents/anticoagulants	B01^¥^	10	11	7, 15	0.0073	78.60
Antidepressants	N06A^β^	4	6	0.4, 17	0.0253	94.90
Antidiabetic	A10^¥^	4	7	0.8, 17	0.0162	77.50
Antiepileptics	N03^¥^	3	4	0.2, 10	0.0029	26.40
Anti-inflammatory and antirheumatic	M01^¥^	5	15	7, 26	0.0166	79.40
Antipsychotics	N05A^β^	4	11	0.2, 32	0.0622	95.60
Cardiovascular	C*	14	16	11, 23	0.0205	90.80
Corticosteroids	H02^¥^	5	2	0.2, 6	0.0066	77.10
Diuretics	C03^¥^	5	6	0.2, 17	0.0365	95.80
Functional gastrointestinal disorders	A03^¥^	3	4	0, 14	0.0183	75.80
Musculoskeletal system	M*	3	9	2.4, 20	0.0142	85.00
Nervous system	N*	6	21	6, 43	0.0784	97.00
Opioids	N02A^β^	5	6	2, 10	0.0045	74.70
Respiratory system-related drugs	R*	4	1	0, 4	0.0061	74.90
Sedatives	N05C^β^	5	16	11, 21	0.0016	33.50

*ATC 1st level—related to mostly body function;

^¥^ATC 2nd level—pharmacological or therapeutic subgroup

^β^ATC 3rd and 4th levels—chemical, pharmacological or therapeutic subgroup

### Meta-regressions to explore heterogeneity in the prevalence of preventable medication harm

Table 3 shows the results of the univariable and multivariable analysis. The univariable analyses showed that the prevalence of preventable medication harm was higher across studies with smaller (≤ 1200 patients) sample sizes (*b* = 0.111, 95% confidence interval 0.064 to 0.159), those carried out in Europe (*b* = 0.031, − 0.0002 to 0.061) and at lower risk of bias (*b* = 0.075, 0.022 to 0.128). The prevalence of preventable harm was lower in children or adolescent-based studies (*b* = − 0.088, − 0.151 to − 0.025) and in studies that assessed medication harm by survey, telephone or spontaneous reporting surveillance systems (*b* = − 0.026, − 0.053 to 0.0003). When compared to general hospitals/internal medicine, healthcare settings involving the elderly (*b* = 0.094, 0.052 to 0.136), emergency departments (*b* = 0.042, 0.011 to 0.073), highly specialised or surgical care (*b* = 0.062, 0.030 to 0.093) or intensive care (b = 0.052, 0.010 to 0.094) showed elevated significant levels of preventable harm. These five variables (sample size, healthcare setting, WHO region, age group, assessment method and low risk of bias) were therefore eligible for inclusion in the multivariable regression analysis.

**Table 3:** Univariable and multivariable meta-regression analysis of prevalence estimation for preventable medication harm

		Univariable			Multivariable		
Variable	No.	Regression coefficient (95% CI)	SE	***P*** value	Regression coefficient (95% CI)	SE	***P*** value
*WHO region:*							
- Asia/other	32	1	–	–	1	–	–
- North America	23	0.018 (−0.014, 0.049)	0.016	0.273	0.03 (−0.02, 0.087)	0.028	0.237
- Europe	26	0.031 (− 0.0002, 0.061)	0.016	**0.051**	0.011 (− 0.045, 0.066)	0.028	0.709
*Drug classification:*							
- Yes	67	1	–	–	–	–	–
- No	14	− 0.011 (− 0.081, 0.059)	0.036	0.760	NA	NA	NA
Sample size*:							
- > 1,200 patients	41	1	–	–	1	–	–
- ≤ 1,200 patients	40	0.111 (0.064, 0.159)	0.024	**< 0.0001**	0.038 (0.016, 0.060)	0.011	**0.036**
*Age group:*							
- Adults	63	1	–	–	1	–	–
- Children/adolescents	18	− 0.088 (− 0.151, − 0.025)	0.032	**0.0060**	− 0.016 (− 0.048, 0.016)	0.016	0.319
*Healthcare setting:*							
- General hospital or internal medicine	29	1	–	–	1	–	–
- Elderly	7	0.094 (0.052, 0.136)	0.021	**< 0.0001**	0.07 (0.023, 0.107)	0.021	**0.002**
- Emergency department	12	0.042 (0.011, 0.073)	0.016	**0.009**	0.028 (− 0.002, 0.058)	0.015	**0.062**
- Highly specialised care or surgical	13	0.062 (0.030, 0.093)	0.016	**0.0001**	0.052 (0.022, 0.082)	0.015	**0.0007**
- ICU	6	0.052 (0.010, 0.094)	0.021	**0.015**	0.038 (− 0.004, 0.080)	0.021	0.073
- Paediatric	9	− 0.008 (− 0.044, 0.028)	0.018	0.663	− 0.0001 (− 0.044, 0.043)	0.022	0.996
- Primary care	4	− 0.009 (− 0.056, 0.039)	0.024	0.720	− 0.014 (− 0.058, 0.031)	0.023	0.547
- Psychiatric	1	− 0.010 (− 0.100, 0.080)	0.046	0.825	− 0.006 (− 0.090, 0.079)	0.043	0.897
*Assessment method:*							
- Medical record review/chart review or observation	54	1	–	–	1	–	–
- Survey, telephone, voluntary (spontaneous) report	27	− 0.026 (− 0.053, 0.0003)	0.014	**0.053**	− 0.021 (− 0.045, 0.004)	0.012	0.099
*Standard method for preventability:*							
- Yes	49	1	–	–	NA	NA	NA
- No	32	− 0.014 (− 0.069, 0.040)	0.028	0.613	NA	NA	NA
*Length of study:*							
- ≤ 6 months	47	1	–	–	NA	NA	NA
- > 6 months	34	−0.026 (−0.080, 0.029)	0.028	0.357	NA	NA	NA
*Design:*							
- Prospective	58	1	–	–	–	–	–
- Retrospective cohort or cross-sectional	23	0.011 (− 0.019, 0.040)	0.015	0.480	NA	NA	NA
*Risk of bias:*							
- High (score < 7)	31	1	–	–	NA	NA	NA
- Low (score ≥ 7)	50	0.075 (0.022, 0.128)	0.027	**0.006**	0.019 (− 0.004, 0.043)	0.012	0.107
**Model fit indices**					*χ*^2^ (6) = 44.228, *P* < 0.001, *R*^2^ = 37.65%		

*SE* standard error, *NA* not applicable

*Medium number of patients = 1200

The multivariable model was statistically significant (*χ*^2^ (6) = 44.228, *P* <  0.001, *R*^2^ = 37.65%) and reduced the *I*^2^ statistic from 99 to 61.3%. Only two of the variables remained statistically significant; studies with low sample sizes (*b* = 0.038, 0.016 to 0.060) and the three healthcare setting groups, including elderly patient care (*b* = 0.07, 0.023 to 0.107), emergency medicine (*b* = 0.028, − 0.002 to 0.058) and highly specialised care (*b* = 0.052, 0.022 to 0.082), were all associated with a higher prevalence of preventable medication harm.

### Publication bias

Additional file 7: Fig. S1 shows some evidence of publication bias as indicated by visual inspections of the funnel plots and by the Egger regression test for small study effects for the primary outcome (bias coefficient for the main analysis 1.39, 95% 0.38 to 3.47, *P* <  0.001). Trim-and-fill method also revealed evidence of publication bias.

## Discussion

This meta-analysis found that preventable medication harm occurs in 3% of patients across medical care settings and that at least a quarter of preventable medication harm is severe or potentially life threatening. In our previous meta-analysis, the prevalence of preventable patient harm (e.g. adverse events due to any type medical errors—not only medication) was 6%, and only one tenth of this harm was severe or potentially life-threatening [50]. Thus, medication harm indeed accounts for half of the overall preventable harm in medical care. The present findings also align closely to a recent narrative meta-review of systematic reviews [51], which looked at the preventability of ADRs in patients receiving acute or ambulatory care—reporting a prevalence rate of 3.13%. However, this earlier narrative overview involved only 37 studies compared to the 81 studies in our meta-analysis, did not include patients in primary care or emergency care settings and did not examine thoroughly the severity of harm, patient age, stage of medication use and high-risk medication groups.

The highest prevalence rate of preventable medication harm was seen in studies based on elderly care units which often involve patients with high comorbidity and hence polypharmacy. The headline policy implication of this study therefore is that mitigation strategies for preventable medication harm are primarily needed for older people with multimorbidity/polypharmacy—which is indeed a key priority area of the WHO safety initiative [16, 52]. A recent review that tried to determine which interventions, alone or in combination, were effective in improving the appropriate use of polypharmacy and reducing medication related harms in older people [53], revealed great uncertainty about the effectiveness of comprehensive medicines reviews of patient prescriptions and urged for further research. Since the last update of this review, a Scottish working group has published a guidance document on polypharmacy, which included a seven-step process for standardised and structured medicines reviews that are holistic, patient-centred and consider non-pharmacological treatments [54], as well as a review of the quality of development of available guidelines to promote appropriate polypharmacy [55]. Further population-based research is needed to evaluate the implementation and effect of these resources on prescribing for older people.

Moreover, medication harm in ICUs [56] and acute specialised care settings involving surgery [57] was associate with higher volumes of preventable medication harm and should, therefore, also be considered as high-risk patient care settings. Evidence concerning the prevalence and severity of preventable medication harm in primary care and psychiatry was scarce. We only found four studies based in primary care, where over 80% of healthcare services are delivered internationally [58], and only one study was identified in psychiatry. It is therefore possible that certain types of preventable harm in psychiatry settings may remain undetected, as these harms often result from multiple interacting errors from violation provoking conditions and latent ‘system’ failures [22, 59]. Thus, there is a need for more research in both these care settings.

The prescribing and monitoring stages of medication use were key sources of preventable harm. The adoption of electronic health records and electronic prescribing has helped avert preventable harm at the prescribing and transcribing stages [14, 60–66], but preventable harm persists across all pathways of medication use. This is most likely due to underlying system flaws that allow individual errors such as those in prescribing or medicines administration to reach the patient and cause serious harm [67]. Human factors play an important role in understanding these system flaws; for instance, there may be a lack of standard procedures for storage of medications that look alike, poor communication between different providers, lack of verification before medication administration and limited involvement of patients in their own care [68–71]. Better safeguarding processes that acknowledge the contribution of human factors and systems thinking are required at the different points of medication use to ensure correct measures are contributing to healthcare improvement efforts [72].

We found that medication groups that gave rise to most preventable medication harm include; central nervous system, cardiovascular, hypnotics and sedatives, anti-inflammatory and antirheumatic, antibiotics and antibacterial drugs. Many of these medication groups have been previously assessed in relation to hazardous prescribing in the past [14, 15]. However, the interventions featured in these studies deliberately focused on potential medication errors rather than preventable harm, and therefore, available evidence has not yet determined whether these interventions will reduce harm to patients. This stresses further the urgency and need to focus on assessment of medication errors and harm in conjunction [13, 71], as well as new ways of reducing medication harms in older people [53].

Most efforts so far originate in developed countries [73], and so the rate of preventable medication harm from less developed countries remains relatively unknown. However, in one study, the preventability of adverse events across 26 hospitals in eight low-and middle-income countries showed an adverse event rate to be around 8%. Of these events, 83% were preventable, while about 30% were associated with death of the patient [74]. Although this is likely to be an underestimate with higher rates of excess death unaccounted for, it shows that patient safety is a much bigger problem in these developing and transitional countries, when judged by the preventability and severity of harm (number of preventable deaths). These results are somewhat contrasting with our results, with the higher rate of preventable harm being found in studies from Europe. However, these findings might reflect the limitations that exist in assessing preventable medication harm in developing countries. Moreover, the prevention of medication harm is often complex and involves improving basic clinical processes and does not simply depend on the provision of more resources [75].

Despite this being the first large meta-analysis to assess the prevalence of preventable medication harm across healthcare settings, there still remain several limitations. First, a considerable proportion of the high heterogeneity remained unexplained in meta-regressions. Several other factors such as differences in systems, procedures and variations in timeframe used to evaluate medication harm remain largely unknown and may be responsible for this unexplained heterogeneity.

Second, whilst we performed exhaustive searches for unpublished studies, the exclusion of non-English written studies and the presence of publication bias that forms of selection bias or system bias is likely [76]. We tried to account for this in the analysis by adjusting for sample size, but some of the causal factors remain unexplained.

Third, studies not reporting data on preventable medication harm were excluded from the analyses. Among the included studies, only 36% of studies provided an analysis of severity and even the classification system used was unclear at times so a pragmatic judgement was made to group some of the severity categories. Furthermore, discussions of causality assessment were limited with few studies reporting the exact assessment tool used, and only 22% of studies provided the stage of medication use, and whilst harm was reported by medication group in 78% of the studies, only one fifth of these provided data at the preventable level. The use of different preventability scales may also incur a level of ‘hindsight bias’, where healthcare professionals may be subject to overestimation of their ability to predict preventable harm events [77, 78]. However, adjustment for this in observation research can be a significant challenge in evidence synthesis.

Finally, more than two thirds of preventable medication harm were examined retrospectively through medical case notes and chart reviews. Although case note reviews are the most universally used method for assessing medication harm to date, patients and healthcare providers suggest that case reviews still lack the robustness to detect diagnostic error and are susceptible to time-delay issues in the absence of regular patient consultation [79]. Combining methods that prospectively uncover preventable harm by use of the ‘failure mode and effect analysis’, ‘structured what-if technique’ [13], pharmacist screening, or patient surveys along with retrospective error detection methods including trigger tools, voluntary reporting systems, root cause analysis or mortality reviews [76, 80] would provide better and more promising approaches for enhancing the detection of preventable medication harm.

## Conclusions

Our study findings confirm that preventable medication-related harm is a frequent and enduring serious problem, causing severe or potentially life-threatening outcomes in over a quarter of all preventable harm cases. A highly problematic healthcare setting was in geriatric care, specialised care settings, intensive care or those in emergency departments. Elderly patients are at greater risk of polypharmacy, and patients in other specialised or emergency medicine settings may be exposed to higher-risk mediation groups. As both are priority areas of the WHO’s safety initiative, we have gone some way in this review to providing a basis of evidence to support future policy developments in this area. Nevertheless, further research is needed in primary care and psychiatry settings where up to 80% of healthcare service is delivered, alongside efforts to better understand where improvements can be made in less-developed countries.

## Supplementary information

**Additional file 1: Table S1.** Moose checklist.

**Additional file 2: Table S2.** Searches.

**Additional file 3: Table S3.** Citations for eligible studies included in meta-analysis.

**Additional file 4: Table S4.** Characteristics of included studies.

**Additional file 5: Table S5.** Critical appraisal ratings for all studies included in review.

**Additional file 6: Table S6.**. Top five most common preventable medication harms by ATC drug classification. % represent prevalence’s.

**Additional file 7: Figure S1.** Funnel plot of preventable medication harm (log-transformed proportion).

## Data Availability

Data and statistical code are available upon request from the corresponding author.
