# Supplementary material for: Preventable medication harm across health care settings: a systematic review and meta-analysis
Source: BMC Med. 2020 Nov 6;18:313. doi: 10.1186/s12916-020-01774-9 (PMC7646069; doi:10.1186/s12916-020-01774-9)
Supplement: Supplementary file 2 — Additional file 2: Table S2. Searches. [file 12916_2020_1774_MOESM2_ESM.docx]

# **Additional file 2: Table S2: Searches**

**Table S2: Medline**

| # ▲ | Searches | Results |
| --- | --- | --- |
| 1 | ((preventable or avoidable or unnecessary or untoward or ameliorable) adj2 (harm or complication* or omission)).mp. | 1897 |
| 2 | exp Medical Errors/cl, mt, pc, st, sn [Classification, Methods, Prevention & Control, Standards, Statistics & Numerical Data] | 26126 |
| 3 | exp medical error/pc or medical error.mp. | 20816 |
| 4 | "Drug-Related Side Effects and Adverse Reactions"/ | 31432 |
| 5 | ((Adverse drug or adverse medication) adj1 (event* or incident or reaction* or effect* or outcome*)).mp. | 21383 |
| 6 | Human error*.mp. | 1869 |
| 7 | ((service* or system* or communication* or organization* or organisation* or treatment or therap* or diagnos*) adj1 (weak* or fail* or error* or mistake* or delay*)).mp. | 136091 |
| 8 | (adverse* adj1 (event* or outcome* or complication* or effect* or reaction*)).mp. | 1899683 |
| 9 | ((psychological or emotional or physical) adj1 (harm or complication*)).mp. | 1283 |
| 10 | patient safety.mp. or Patient Safety/ | 36658 |
| 11 | (death* or accident or serious incident* or injur* or adverse event*).mp. | 1942696 |
| 12 | 10 and 11 | 6326 |
| 13 | (never event* or near miss*).mp. | 2184 |
| 14 | (iatrogenic adj (harm or injur* or complication*)).mp. | 3254 |
| 15 | Patient Harm/ or patient harm.mp. | 1253 |
| 16 | Diagnostic Errors/ | 37308 |
| 17 | (preventable or avoidable or unnecessary or untoward or ameliorable).mp. | 84507 |
| 18 | 2 or 3 or 4 or 5 or 6 or 7 or 8 or 9 or 12 or 13 or 14 or 15 or 16 | 2041058 |
| 19 | 17 and 18 | 17951 |
| 20 | 1 or 19 | 18958 |
| 21 | Prevalence/ or prevalence.mp. | 606843 |
| 22 | incidence.mp. or Incidence/ | 746452 |
| 23 | Epidemiologic Studies/ | 8241 |
| 24 | exp Case-Control Studies/ | 1063053 |
| 25 | (epidemiologic* adj (study or studies)).mp. | 79712 |
| 26 | case control.mp. | 307790 |
| 27 | exp Cohort Studies/ | 1967485 |
| 28 | Cross-Sectional Studies/ | 320859 |
| 29 | (cohort adj (study or studies)).mp. | 347674 |
| 30 | Cohort analy*.mp. | 7196 |
| 31 | (follow up adj (study or studies)).mp. | 653458 |
| 32 | longitudinal.mp. | 254760 |
| 33 | Retrospective.mp. | 882299 |
| 34 | Prospective.mp. | 712179 |
| 35 | (observ* adj1 (study or studies)).mp. | 139452 |
| 36 | (analytical adj (study or studies)).mp. | 3818 |
| 37 | (comparative adj (study or studies)).mp. | 1893348 |
| 38 | (evaluation adj (study or studies)).mp. | 375351 |
| 39 | Meta-analysis/ | 112011 |
| 40 | ((Systematic or narrative) adj review).mp. | 141517 |
| 41 | Clinical Trial/ or Randomized Controlled Trial/ | 808081 |
| 42 | or/23-41 | 5116082 |
| 43 | 20 and 42 | 7639 |
| 44 | 21 or 22 | 1277364 |
| 45 | 20 and 44 | 3714 |
| 46 | 43 or 45 | 9200 |
| 47 | limit 46 to (english language and yr="2000 -Current") | 6902 |

**Table 3: Embase**

| # ▲ | Searches | Results |
| --- | --- | --- |
| 1 | ((preventable or avoidable or unnecessary or untoward or ameliorable) adj2 (harm or complication* or omission)).mp. | 3322 |
| 2 | exp medical error/pc or medical error.mp. | 22471 |
| 3 | "Drug-Related Side Effects and Adverse Reactions"/ | 169060 |
| 4 | ((Adverse drug or adverse medication) adj1 (event* or incident or reaction* or effect* or outcome*)).mp. | 1451288 |
| 5 | Human error*.mp. | 3605 |
| 6 | ((service* or system* or communication* or organization* or organisation* or treatment or therap* or diagnos*) adj1 (weak* or fail* or error* or mistake* or delay*)).mp. | 308862 |
| 7 | (adverse* adj1 (event* or outcome* or complication* or effect* or reaction*)).mp. | 612810 |
| 8 | ((psychological or emotional or physical) adj1 (harm or complication*)).mp. | 2153 |
| 9 | patient safety.mp. or Patient Safety/ | 129226 |
| 10 | (death* or accident or serious incident* or injur* or adverse event*).mp. | 3242096 |
| 11 | 9 and 10 | 27520 |
| 12 | (never event* or near miss*).mp. | 4102 |
| 13 | (iatrogenic adj (harm or injur* or complication*)).mp. | 5484 |
| 14 | Patient Harm/ or patient harm.mp. | 3268 |
| 15 | Diagnostic Errors/ | 52477 |
| 16 | (preventable or avoidable or unnecessary or untoward or ameliorable).mp. | 137038 |
| 17 | Epidemiologic Studies/ | 207173 |
| 18 | exp Case-Control Studies/ | 171116 |
| 19 | (epidemiologic* adj (study or studies)).mp. | 109450 |
| 20 | case control.mp. | 228771 |
| 21 | exp Cohort Studies/ | 559046 |
| 22 | Cross-Sectional Studies/ | 212042 |
| 23 | (cohort adj (study or studies)).mp. | 295075 |
| 24 | Cohort analy*.mp. | 561600 |
| 25 | (follow up adj (study or studies)).mp. | 65816 |
| 26 | longitudinal.mp. | 360025 |
| 27 | Retrospective.mp. | 1209495 |
| 28 | Prospective.mp. | 1026701 |
| 29 | (observ* adj1 (study or studies)).mp. | 247539 |
| 30 | (analytical adj (study or studies)).mp. | 7090 |
| 31 | (comparative adj (study or studies)).mp. | 906893 |
| 32 | (evaluation adj (study or studies)).mp. | 51325 |
| 33 | Meta-analysis/ | 182621 |
| 34 | ((Systematic or narrative) adj review).mp. | 312230 |
| 35 | Clinical Trial/ or Randomized Controlled Trial/ | 1272525 |
| 36 | 2 or 3 or 4 or 5 or 6 or 7 or 8 or 11 or 12 or 13 or 14 or 15 | 2131049 |
| 37 | 16 and 36 | 21889 |
| 38 | 1 or 37 | 24286 |
| 39 | or/17-35 | 5373004 |
| 40 | 38 and 39 | 8839 |
| 41 | limit 40 to (english language and yr="2000 -Current") | 6568 |

**Table 4: PsychInfo**

| # ▲ | Searches | Results |
| --- | --- | --- |
| 1 | ((preventable or avoidable or unnecessary or untoward or ameliorable) adj2 (harm or complication* or omission)).mp. | 169 |
| 2 | medical error.mp. | 300 |
| 3 | ((Adverse drug or adverse medication) adj1 (event* or incident or reaction* or effect* or outcome*)).mp. | 1617 |
| 4 | Human error*.mp. | 950 |
| 5 | ((service* or system* or communication* or organization* or organisation* or treatment or therap* or diagnos*) adj1 (weak* or fail* or error* or mistake* or delay*)).mp. | 8982 |
| 6 | (adverse* adj1 (event* or outcome* or complication* or effect* or reaction*)).mp. | 32125 |
| 7 | ((psychological or emotional or physical) adj1 (harm or complication*)).mp. | 1503 |
| 8 | patient safety.mp. or Patient Safety/ | 4756 |
| 9 | (death* or accident or serious incident* or injur* or adverse event*).mp. | 212593 |
| 10 | 8 and 9 | 941 |
| 11 | (never event* or near miss*).mp. | 528 |
| 12 | (iatrogenic adj (harm or injur* or complication*)).mp. | 145 |
| 13 | Patient Harm/ or patient harm.mp. | 182 |
| 14 | (preventable or avoidable or unnecessary or untoward or ameliorable).mp. | 12099 |
| 15 | 2 or 3 or 4 or 5 or 6 or 7 or 10 or 11 or 12 or 13 | 45610 |
| 16 | 14 and 15 | 640 |
| 17 | 1 or 16 | 774 |
| 18 | limit 17 to (english language and yr="2000 -Current") | 697 |

**Table 5: HMIC**

| # ▲ | Searches | Results |
| --- | --- | --- |
| 1 | ((preventable or avoidable or unnecessary or untoward or ameliorable) adj2 (harm or complication* or omission)).mp. | 94 |
| 2 | Medical error.mp. | 125 |
| 3 | ((Adverse drug or adverse medication) adj1 (event* or incident or reaction* or effect* or outcome*)).mp. | 948 |
| 4 | Human error*.mp. | 224 |
| 5 | ((service* or system* or communication* or organization* or organisation* or treatment or therap* or diagnos*) adj1 (weak* or fail* or error* or mistake* or delay*)).mp. | 707 |
| 6 | (adverse* adj1 (event* or outcome* or complication* or effect* or reaction*)).mp. | 3114 |
| 7 | ((psychological or emotional or physical) adj1 (harm or complication*)).mp. | 53 |
| 8 | patient safety.mp. or Patient Safety/ | 4666 |
| 9 | (death* or accident or serious incident* or injur* or adverse event*).mp. | 19272 |
| 10 | 8 and 9 | 1055 |
| 11 | (never event* or near miss*).mp. | 162 |
| 12 | (iatrogenic adj (harm or injur* or complication*)).mp. | 37 |
| 13 | Patient Harm/ or patient harm.mp. | 109 |
| 14 | (preventable or avoidable or unnecessary or untoward or ameliorable).mp. | 2694 |
| 15 | 2 or 3 or 4 or 5 or 6 or 7 or 10 or 11 or 12 or 13 | 5085 |
| 16 | 14 and 15 | 307 |
| 17 | 1 or 16 | 359 |
| 18 | limit 17 to yr="2000 -Current" | 298 |
