# Supplementary material for: Preventable medication harm across health care settings: a systematic review and meta-analysis
Source: BMC Med. 2020 Nov 6;18:313. doi: 10.1186/s12916-020-01774-9 (PMC7646069; doi:10.1186/s12916-020-01774-9)
Supplement: Supplementary file 3 — Additional file 3: Table S3. Citations for eligible studies included in meta-analysis. [file 12916_2020_1774_MOESM3_ESM.docx]

# **Additional file 3: Table S3: Eligible studies included in meta-analysis**

| **Citation** |
| --- |
| Ahern F, Sahm LJ, Lynch D, McCarthy S. Determining the frequency and preventability of adverse drug reaction-related admissions to an Irish University Hospital: a cross-sectional study. *Emergency Medicine Journal.* 2014;31(1):24-29. |
| Alam K, Shakya R, Ojha P. Reporting adverse drug reactions among hospitalized medical patients: A prospective study from tertiary care hospital in Western Nepal. *Nepal Journal of Epidemiology.* 2014;4(1):330-336. |
| Aljadhey H, Mahmoud MA, Mayet A, et al. Incidence of adverse drug events in an academic hospital: a prospective cohort study. *International Journal for Quality in Health Care.* 2013;25(6):648-655. |
| Alsbou M. Incidence of adverse drug reactions in alkarak hospital: A pilot study. *Jordan Medical Journal.* 2010;44(4):442-446. |
| Alsbou M, Alzubiedi S, Alzobi H, et al. Adverse drug reactions experience in a teaching hospital in Jordan. *International Journal of Clinical Pharmacy.* 2015;37(6):1188-1193. |
| Al-Tajir GK, Kelly WN. Epidemiology, comparative methods of detection, and preventability of adverse drug events. *Annals of Pharmacotherapy.* 2005;39(7-8):1169-1174. |
| Ayani N, Sakuma M, Morimoto T, et al. The epidemiology of adverse drug events and medication errors among psychiatric inpatients in Japan: the JADE study. *BMC Psychiatry.* 2016;16(1):303-303. |
| Benard-Laribiere A, Miremont-Salame G, Perault-Pochat M-C, Noize P, Haramburu F, centres ESGobotFnop. Incidence of hospital admissions due to adverse drug reactions in France: the EMIR study. *Fundamental & Clinical Pharmacology.* 2015;29(1):106-111. |
| Benkirane R, Pariente A, Achour S, Ouammi L, Azzouzi A, Soulaymani R. Prevalence and preventability of adverse drug events in a teaching hospital: a cross-sectional study. *Eastern Mediterranean Health Journal.* 2009;15(5):1145-1155. |
| Benkirane RR, R RA, Haimeur CC, et al. Incidence of adverse drug events and medication errors in intensive care units: a prospective multicenter study. *Journal of patient safety.* 2009;5(1):16-22. |
| Buckley MS, Erstad BL, Kopp BJ, Theodorou AA, Priestley G. Direct observation approach for detecting medication errors and adverse drug events in a pediatric intensive care unit. *Pediatric Critical Care Medicine.* 2007;8(2):145-152. |
| Calderon-Ospina C, Bustamante-Rojas C. The DoTS classification is a useful way to classify adverse drug reactions: a preliminary study in hospitalized patients. *International Journal of Pharmacy Practice.* 2010;18(4):230-235. |
| Carayon P, Wetterneck TB, Cartmill R, et al. Characterising the complexity of medication safety using a human factors approach: an observational study in two intensive care units. *BMJ quality & safety.* 2014;23(1):56-65. |
| Castro I, Guardiola JM, Tuneu L, Sala ML, Faus MJ, Mangues MA. Drug-related visits to the emergency department in a Spanish university hospital. *International Journal of Clinical Pharmacy.* 2013;35(5):727-735. |
| Chan M, Nicklason F, Vial JH. Adverse drug events as a cause of hospital admission in the elderly. *Internal Medicine Journal.* 2001;31(4):199-205. |
| Chanie Eshetie T, Hailemeskel B, Mekonnen N, Paulos G, Mekonnen AB, Girma T. Adverse drug events in hospitalized children at Ethiopian University Hospital: a prospective observational study. *Bmc Pediatr.* 2015;15:83. |
| Chen YC, Fan JS, Hsu TF, et al. Detection of patients presenting with adverse drug events in the emergency department. *Internal Medicine Journal.* 2012;42(6):651-657. |
| Damen L, Basheti I. Preventability analysis of adverse drug reactions in a Jordanian hospital: a prospective observational study. *International Journal of Clinical Pharmacy.* 2019;41. |
| Damen NL, Baines R, Wagner C, Langelaan M. Medication-related adverse events during hospitalization: a retrospective patient record review study in The Netherlands. *Pharmacoepidemiology & Drug Safety.* 2017;26(1):32-39. |
| Davies EC, Green CF, Mottram DR, Rowe PH, Pirmohamed M. Emergency re-admissions to hospital due to adverse drug reactions within 1 year of the index admission. *Br J Clin Pharmacol.* 2010;70(5):749-755. |
| Davies EC, Green CF, Taylor S, Williamson PR, Mottram DR, Pirmohamed M. Adverse drug reactions in hospital in-patients: a prospective analysis of 3695 patient-episodes. *PLoS ONE [Electronic Resource].* 2009;4(2):e4439. |
| De Boer M, Boeker EB, Ramrattan MA, et al. Adverse drug events in surgical patients: An observational multicentre study. *International Journal of Clinical Pharmacy.* 2013;35(5):744-752. |
| Dequito AB, Mol PGM, van Doormaal JE, et al. Preventable and non-preventable adverse drug events in hospitalized patients: a prospective chart review in the Netherlands. *Drug Safety.* 2011;34(11):1089-1100. |
| Easton-Carter KL, Chapman CB, Brien JAE. Adverse drug reactions in paediatrics: Are we getting the full picture? *Journal of Pharmacy Practice and Research.* 2003;33(2):106-110. |
| Farcas A, Sinpetrean A, Mogosan C, et al. Adverse drug reactions detected by stimulated spontaneous reporting in an internal medicine department in Romania. *European Journal of Internal Medicine.* 2010;21(5):453-457. |
| Forster AJ, Halil RB, Tierney MG. Pharmacist surveillance of adverse drug events. *American Journal of Health-System Pharmacy.* 2004;61(14):1466-1472. |
| Forster AJ, Murff HJ, Peterson JF, Gandhi TK, Bates DW. Adverse drug events occurring following hospital discharge. *Journal of general internal medicine.* 2005;20(4):317-323. |
| Franceschi M, Scarcelli C, Niro V, et al. Prevalence, clinical features and avoidability of adverse drug reactions as cause of admission to a geriatric unit: a prospective study of 1756 patients. *Drug Safety.* 2008;31(6):545-556. |
| Gallagher RM, Mason JR, Bird KA, et al. Adverse drug reactions causing admission to a paediatric hospital. *PLoS ONE [Electronic Resource].* 2012;7(12):e50127. |
| Gandhi TK, Weingart SN, Borus J, et al. Adverse drug events in ambulatory care. *New England Journal of Medicine.* 2003;348(16):1556-1564. |
| Geer MI, Koul PA, Tanki SA, Shah MY. Frequency, types, severity, preventability and costs of Adverse Drug Reactions at a tertiary care hospital. *J Pharmacol Tox Met.* 2016;81:323-334. |
| Grenouillet-Delacre M, Verdoux H, Moore N, et al. Life-threatening adverse drug reactions at admission to medical intensive care: a prospective study in a teaching hospital. *Intensive Care Medicine.* 2007;33(12):2150-2157. |
| Gurwitz JH, Field TS, Avorn J, et al. Incidence and preventability of adverse drug events in nursing homes. *American Journal of Medicine.* 2000;109(2):87-94. |
| Gurwitz JH, Field TS, Harrold LR, et al. Incidence and preventability of adverse drug events among older persons in the ambulatory setting. *Jama.* 2003;289(9):1107-1116. |
| Gurwitz JH, Field TS, Judge J, et al. The incidence of adverse drug events in two large academic long-term care facilities. *American Journal of Medicine.* 2005;118(3):251-258. |
| Haile DB, Ayen WY, Tiwari P. Prevalence and assessment of factors contributing to adverse drug reactions in wards of a tertiary care hospital, India. *Ethiop J Health Sci.* 2013;23(1):39-48. |
| Hamilton H, Gallagher P, Ryan C, Byrne S, O'Mahony D. Potentially inappropriate medications defined by STOPP criteria and the risk of adverse drug events in older hospitalized patients. *Archives of internal medicine.* 2011;171(11):1013-1019. |
| Hardmeier B, Braunschweig S, Cavallaro M, et al. Adverse drug events caused by medication errors in medical inpatients. *Swiss medical weekly.* 2004;134(45-46):664-670. |
| Harkanen M, Kervinen M, Ahonen J, Voutilainen A, Turunen H, Vehvilainen-Julkunen K. Patient-specific risk factors of adverse drug events in adult inpatients - evidence detected using the Global Trigger Tool method. *Journal of Clinical Nursing.* 2015;24(3-4):582-591. |
| Harugeri A, Parthasarathi G, Ramesh M, Guido S, Basavanagowdappa H. Frequency and nature of adverse drug reactions in elderly in-patients of two Indian medical college hospitals. *Journal of Postgraduate Medicine.* 2011;57(3):189-195. |
| Hoonhout LHF, de Bruijne MC, Wagner C, Asscheman H, van der Wal G, van Tulder MW. Nature, occurrence and consequences of medication-related adverse events during hospitalization: a retrospective chart review in the Netherlands. *Drug Safety.* 2010;33(10):853-864. |
| Howard RL, Avery AJ, Howard PD, Partridge M. Investigation into the reasons for preventable drug related admissions to a medical admissions unit: observational study. *Qual Saf Health Care.* 2003;12(4):280-285. |
| Hug BL, Witkowski DJ, Sox CM, et al. Adverse drug event rates in six community hospitals and the potential impact of computerized physician order entry for prevention. *Journal of general internal medicine.* 2010;25(1):31-38. |
| Ithnin M, Latif Z, Syaiful A, Rani M, Nor Aripin K. Incidence of adverse drug reactions in a paediatric ward of a Malaysian hospital: A prospective observational study. *Tropical Journal of Pharmaceutical Research.* 2018;17:1423-1431. |
| Jha AK, Kuperman GJ, Rittenberg E, Teich JM, Bates DW. Identifying hospital admissions due to adverse drug events using a computer-based monitor. *Pharmacoepidem Dr S.* 2001;10(2):113-119. |
| Jonsson AK, Hakkarainen KM, Spigset O, Druid H, Hiselius A, Hagg S. Preventable drug related mortality in a Swedish population. *Pharmacoepidem Dr S.* 2010;19(2):211-215. |
| Kaushal R, Bates DW, Landrigan C, et al. Medication errors and adverse drug events in pediatric inpatients. *Jama.* 2001;285(16):2114-2120. |
| Kaushal R, Goldmann DA, Keohane CA, et al. Adverse drug events in pediatric outpatients. *Ambulatory Pediatrics.* 2007;7(5):383-389. |
| Klopotowska JE, Wierenga PC, Stuijt CCM, et al. Adverse drug events in older hospitalized patients: results and reliability of a comprehensive and structured identification strategy. *PLoS ONE [Electronic Resource].* 2013;8(8):e71045. |
| Kopp BJ, Erstad BL, Allen ME, Theodorou AA, Priestley G. Medication errors and adverse drug events in an intensive care unit: direct observation approach for detection. *Critical Care Medicine.* 2006;34(2):415-425. |
| Kunac DL, Kennedy J, Austin N, Reith D. Incidence, preventability, and impact of Adverse Drug Events (ADEs) and potential ADEs in hospitalized children in New Zealand: a prospective observational cohort study. *Paediatric Drugs.* 2009;11(2):153-160. |
| Lagnaoui R, Moore N, Fach J, Longy-Boursier M, Begaud B. Adverse drug reactions in a department of systemic diseases-oriented internal medicine: prevalence, incidence, direct costs and avoidability. *Eur J Clin Pharmacol.* 2000;56(2):181-186. |
| Laroche M-L, Perault-Pochat M-C, Ingrand I, et al. Adverse drug reactions in patients with Alzheimer's disease and related dementia in France: a national multicentre cross-sectional study. *Pharmacoepidem Dr S.* 2013;22(9):952-960. |
| Ligi I, Arnaud F, Jouve E, Tardieu S, Sambuc R, Simeoni U. Iatrogenic events in admitted neonates: a prospective cohort study. *Lancet.* 2008;371(9610):404-410. |
| Lopez L, Weissman JS, Schneider EC, Weingart SN, Cohen AP, Epstein AM. Disclosure of hospital adverse events and its association with patients' ratings of the quality of care. *Archives of internal medicine.* 2009;169(20):1888-1894. |
| Lovborg H, Eriksson LR, Jonsson AK, Bradley T, Hagg S. A prospective analysis of the preventability of adverse drug reactions reported in Sweden. *European Journal of Clinical Pharmacology.* 2012;68(8):1183-1189. |
| Meier F, Maas R, Sonst A, et al. Adverse drug events in patients admitted to an emergency department: an analysis of direct costs. *Pharmacoepidemiology & Drug Safety.* 2015;24(2):176-186. |
| Miller GC, Britth HC, Valenti L. Adverse drug events in general practice patients in Australia. *Medical Journal of Australia.* 2006;184(7):321-324. |
| Morimoto T, Sakuma M, Matsui K, et al. Incidence of adverse drug events and medication errors in Japan: the JADE study. *Journal of general internal medicine.* 2011;26(2):148-153. |
| Olivier P, Boulbes O, Tubery M, Lauque D, Montastruc J-L, Lapeyre-Mestre M. Assessing the feasibility of using an adverse drug reaction preventability scale in clinical practice: a study in a French emergency department. *Drug Safety.* 2002;25(14):1035-1044. |
| Park S, In Y, Suh GY, Sohn K, Kim E. Evaluation of adverse drug reactions in medical intensive care units. *European Journal of Clinical Pharmacology.* 2013;69(1):119-131. |
| Patel KJ, Kedia MS, Bajpai D, Mehta SS, Kshirsagar NA, Gogtay NJ. Evaluation of the prevalence and economic burden of adverse drug reactions presenting to the medical emergency department of a tertiary referral centre: a prospective study. *BMC Clinical Pharmacology.* 2007;7:8. |
| Peyriere H, Cassan S, Floutard E, et al. Adverse drug events associated with hospital admission. *The Annals of pharmacotherapy.* 2003;37(1):5-11. |
| Phillips AL, Nigro O, Macolino KA, et al. Hospital admissions caused by adverse drug events: an Australian prospective study. *Australian Health Review.* 2014;38(1):51-57. |
| Pirmohamed M, James S, Meakin S, et al. Adverse drug reactions as cause of admission to hospital: prospective analysis of 18 820 patients. *BMJ.* 2004;329(7456):15-19. |
| Pourseyed S, Fattahi F, Pourpak Z, et al. Adverse drug reactions in patients in an Iranian department of internal medicine. *Pharmacoepidemiology & Drug Safety.* 2009;18(2):104-110. |
| Rachana J, Shastry C, Mateti V, Sharma R, Up N, Chand S. INCIDENCE AND ASSOCIATED FACTORS OF ADVERSE DRUG REACTIONS IN GENERAL MEDICINE DEPARTMENT OF A TERTIARY CARE TEACHING HOSPITAL. *INTERNATIONAL JOURNAL OF ADVANCES IN PHARMACEUTICAL RESEARCH.* 2019;11:177-184. |
| Remesh A, Balan A, Gnanadurai A. A cross-sectional study of surveillance of adverse drug reactions in inpatient departments of a tertiary care hospital. *J Basic Clin Physiol Pharmacol.* 2014;25(1):125-130. |
| Rothschild JM, Mann K, Keohane CA, et al. Medication safety in a psychiatric hospital. *General Hospital Psychiatry.* 2007;29(2):156-162. |
| Sakuma M, Ida H, Nakamura T, et al. Adverse drug events and medication errors in Japanese paediatric inpatients: a retrospective cohort study. *BMJ quality & safety.* 2014;23(10):830-837. |
| Schade CP, Hannah K, Ruddick P, Starling C, Brehm J. Improving self-reporting of adverse drug events in a West Virginia hospital. *American journal of medical quality : the official journal of the American College of Medical Quality.* 2006;21(5):335-341. |
| Senst BL, Achusim LE, Genest RP, et al. Practical approach to determining costs and frequency of adverse drug events in a health care network. *American journal of health-system pharmacy : AJHP : official journal of the American Society of Health-System Pharmacists.* 2001;58(12):1126-1132. |
| Sriram S, Ghasemi A, Ramasamy R, et al. Prevalence of adverse drug reactions at a private tertiary care hospital in south India. *Journal of Research in Medical Sciences.* 2011;16(1):16-25. |
| Sundaran S, Udayan A, Hareendranath K, et al. Study on the Classification, Causality, Preventability and Severity of Adverse Drug Reaction Using Spontaneous Reporting System in Hospitalized Patients. *Pharmacy (Basel, Switzerland).* 2018;6(4). |
| Takata GS, Mason W, Taketomo C, Logsdon T, Sharek PJ. Development, testing, and findings of a pediatric-focused trigger tool to identify medication-related harm in US children's hospitals. *Pediatrics.* 2008;121(4):e927-935. |
| Tangiisuran B, Davies JG, Wright JE, Rajkumar C. Adverse drug reactions in a population of hospitalized very elderly patients. *Drug Aging.* 2012;29(8):669-679. |
| van der Hooft CS, Dieleman JP, Siemes C, et al. Adverse drug reaction-related hospitalisations: a population-based cohort study. *Pharmacoepidemiol Drug Saf.* 2008;17(4):365-371. |
| Woo SA, Cragg A, Wickham ME, et al. Preventable adverse drug events: Descriptive epidemiology. *Br J Clin Pharmacol.* 2020;86(2):291-302. |
| Zandieh SO, Goldmann DA, Keohane CA, Yoon C, Bates DW, Kaushal R. Risk factors in preventable adverse drug events in pediatric outpatients. *Journal of Pediatrics.* 2008;152(2):225-231. |
| Zed PJ, Abu-Laban RB, Balen RM, et al. Incidence, severity and preventability of medication-related visits to the emergency department: a prospective study. *CMAJ Canadian Medical Association Journal.* 2008;178(12):1563-1569. |
