# Supplementary material for: Preventable medication harm across health care settings: a systematic review and meta-analysis
Source: BMC Med. 2020 Nov 6;18:313. doi: 10.1186/s12916-020-01774-9 (PMC7646069; doi:10.1186/s12916-020-01774-9)
Supplement: Supplementary file 4 — Additional file 4: Table S4. Characteristics of included studies. [file 12916_2020_1774_MOESM4_ESM.docx]

# **Additional file 4: Table S4: Characteristics of included studies**

| **Study** | **Sample size** | **Country** | **Design** | **Setting** | **Population** | **Age** | **Length of study** | **ADEs by drug classification** | **Standard preventability criteria used** | **ADEs according to stages of medication** |
| --- | --- | --- | --- | --- | --- | --- | --- | --- | --- | --- |
| Ahern 2014 | 1258 | Ireland | Prospective | Emergency Department | Patients admitted to ED | Mean (sd) = 68.8 (18.0) | 4 weeks | Yes (ATC classification index) | Hallas K score | NR |
| Al Damen 2019 | 350 | Jordan | Prospective | Surgical and medical wards | Patients who were above the age of 18 years, have been taking at least one medication during hospitalization and admitted for at least 24 hrs | Mean = 60.2 (SD 20.8) | 4 months | Yes | Shumock and Thornton scale | NR |
| Alam 2014 | 1105 | Western Nepal | Cross-sectional | Hospital | Patients who stayed at least 24 hours and used at least one medicine | NR, but 31-69 | 6 months | Yes (system affected by ADRs (top 8) | Shumock and Thornton scale | Category and route of administration of drugs causing ADRs: In the study 27 (52.94%) ADRs were occurred after administration of oral drugs while 24 (47.06%) cases occurred by parenteral drugs. |
| Aljadhey 2013 | 977 | Saudi Arabia | Prospective | Hospital | Patients admitted to 8 medical, 5 surgical and 4 intensive care units | Mean (sd) = 48.9 (20.4) | 4 months | Yes (top 12) | Other | Yes |
| Alsbou 2010 | 200 | Jordan | Prospective | Hospital | Patients admitted to internal medicine/ICU over a 4-week period | Median = 45 | 4 weeks | NR - specific drugs reported | Hallas K score | NR |
| Alsbou 2015 | 2000 | Jordan | Cross-sectional | Hospital | Patients that developed/suffered an ADR | Mean = 36 (3 months to 76 years | 11 months | Yes | Shumock and Thornton scale | NR |
| Al-Tajir 2005 | 5235 | UAEs | Prospective | Hospital | Patients in urban tertiary care hospital with 360 beds and multiple specialties | 46.7% were adult patients | 12 months | Yes | Shumock and Thornton scale | Yes (identification through different sources provided) |
| Ayani 2016 | 1234 | Japan | Retrospective cohort | Psychiatric hospital | Inpatients with 22,733 patient-days in a psychiatric hospital and psychiatric units | Mean = 56 (SD 22) | 12 months | Yes | NR | Yes |
| Benkirane 2009a | 1390 | Morocco | Cross-sectional | Hospitals | Adult and paediatric patients in medical/surgical intensive care units for 3 months | Mean = 63.3 | 5 days | Yes | Shumock and Thornton scale | Yes (MD, ICU, ED and surgical dep) |
| Benkirane 2009b | 696 | Morocco | Prospective | Hospital | Adult and paediatric patients in medical/surgical intensive care units for 3 months | Mean = 44.3 (SD 15.4) | 3 months | Yes | NR | Yes (prescribing, admin, transcription, dispensing) |
| Bernad-Laribiere 2015 | 2692 | France | Prospective | Hospital | All patients admitted during a 2-week period in public hospitals | Mean of three groups that have similar samples, 32 years | 2 weeks | Yes (not presented in table) | Oliver et al, scale | NR |
| Buckley 2007 | 357 | US | Prospective | Medical/surgical ICU | Patients with medication error reports | Median = 69 (IQR: 45 to 83) | 6 months | NR | Hallas K score | Yes (prescribing, Transcription, dispensing, admin) |
| Calderon-Ospina 2010 | 104 | Colombia | Cross-sectional | Hospital | All adult patients admitted | Mean = 6.6 (1.2) | 1 month | NR | Shumock and Thornton scale | NR |
| Carayon 2014 | 1805 | US | Cross-sectional | Intensive Care | All adult inpatients Consecutive ICU patient admissions | age distribution, 53.8%(34.8–72.1%) of patients with ADRs were younger than 65years old, and 46.2% (27.9–65.2%) of patients were 65 years or older. | 6 months | NR | NR | Yes |
| Castro 2013 | 588 | Spain | Cross-sectional | Emergency Department | Emergency department patients’ Consecutive ICU patient admissions | Mean = 61 (SD 16) | 3 months | Yes | Baena et al 2013 | NR |
| Chan 2001 | 240 | Australia | Cross-sectional | Hospital | Acute, unplanned, emergency admissions to medical wards of patients who were 75 years or older | Mean = 54.3 (21.6) | 8 weeks | Yes | Hallas K score | NR |
| Chanie Eshetie 2015 | 634 | Ethiopia | Prospective | Hospital | Hospital providing both outpatient and inpatient paediatric services for children less than 14 years | Mean = 81.8 (range, 75-94) | 4 months | Yes | Shumock and Thornton scale | Yes |
| Chen 2012 | 58569 | Taiwan | Prospective | Hospital | Patients 18 years and older presenting to the ED | Mean = 2.9 years | 12 months | Yes | Shumock and Thornton scale | NR |
| Damen 2017 | 8071 | US | Retrospective | Hospital | Patients 18 years and older presenting to the ED | 65.3% above 65 years, 34.7% below 65 | 3 years | Yes | Yes (adapted scale) | Yes |
| Davies 2009 | 3695 | UK | Prospective | Emergency Department | Patients admitted to 12 wards | Mean = 60.2 (SD 20.8) | 6 months | Yes | Hallas K score | NR |
| Davies 2010 | 290 | UK | Prospective | Hospital | Patients admitted to 12 wards | Mean = 62.5 (43-78) | 12 months | Yes | Hallas K score | NR |
| de Boer 2013 | 567 | Holland | Prospective | Surgery | Surgical patients from 3 hospitals with a hospital stay longer than 48 h | Mean = 62 (42-76) | 4 months | Yes | Unclear | NR |
| Dequito 2011 | 609 | Netherlands | Prospective | Hospital | Patients admitted to two Dutch hospitals | Mean = 62 (14.5) | 2 years | Yes | Adapted scale | NR |
| Easton 2003 | 16187 | Australia | Retrospective | Hospital | This multicentre study involved paediatric patients (age < 18 years) at three hospitals | Mean = 68.7 (18.4), model 2) | 4 - 29 weeks | Yes (in text) | Shumock and Thornton scale | NR |
| Farcas 2010 | 1854 | Romania | Prospective | Internal Medicine | NR | Paediatric population (under 18) | 12 months | Yes | French scale (lmbs et al.) | NR |
| Farcas 2014 | 6605 | Romania | Prospective | Internal Medicine | All persons aged 65 or older receiving health care services | Median = 59 (25-92) | 30 months | Yes | Tailored scale similar to Shumock and lmbs | NR |
| Forster 2004 | 543 | Canada | Prospective | Hospital | All patients admitted to the general medicine service | Median = 65.4 (13.2) | 1 month | Yes | Bates | NR |
| Forster 2005 | 400 | US | Prospective | Hospital | Consecutive patients discharged home from the general medical service | Median = 71 (54-81) | 3 months | Yes | Bates | NR |
| Franceschi 2008 | 1756 | Italy | Prospective | Geriatric | All the patients aged ≥65 years admitted to the Geriatric unit | Mean = 57 (17) | 13 month | Yes | Hallas and Gurwitz | NR |
| Gallagher 2012 | 6821 | UK | Prospective | Paediatric hospital | Patients admitted acutely to a paediatric hospital | Mean = 77.2 (7.6) | 12 months | Yes | Hallas | Yes |
| Gandhi 2003 | 661 | US | Prospective | Primary Care | Outpatients older than 18 years | Median = 6 (ADR) | 4 weeks | Yes | Folli et al 1987 | NR |
| Geer 2016 | 5482 | India | Prospective | Internal Medicine | Adult patients admitted in Internal Medicine in-patient department | Mean = 52 (range 19-100) | 270 days | Yes | Hallas | Yes |
| Grenouillet-Delacre 2007 | 405 | France | Prospective | Intensive Care | All patients aged over 15 years and who had received documented drug treatment | Mean = 62 (2.34) | 6 months | Yes | Bates | Yes |
| Gurwitz 2000 | 27617 | US | Retrospective | Hospital | Medicare enrolees receiving medical care | Mean = 64 (17) (with ADR) | 12 months | Yes | Bates | NR |
| Gurwitz 2003 | 2916 | US | Prospective | Long-term Care Facility in nursing homes | All long-term care residents of 18 community-based nursing homes | Mean = 84 (9) | 12 months | Yes | Bates | NA |
| Gurwitz 2005 | 1247 | Canada | Prospective | Long-term Care Facility | All long-stay residents of two academic long-term care facilities | Mean = 74.7 (6.7) | 9 months | Yes | Bates | NR |
| Haile 2013 | 1033 | India | Prospective | Hospital | Patients admitted to hospital for any kind of treatment | Mean = 86 (8) | 8 months | Yes | Shumock and Thornton scale | NR |
| Hamilton 2011 | 600 | Ireland | Prospective | Hospital | Consecutive patients 65 years or older who were admitted with acute illness | Median = 61 (49-68.3) | 4 months | No | Hallas | NR |
| Hardmeier 2004 | 6383 | Switzerland | Prospective | Hospital | Patients with ADE or ADE related hospital admissions | Median = 79 (73-84) with ADE | 4 years | Yes | Unclear | NR |
| Harkanen 2015 | 463 | Finland | Retrospective | Hospital | Randomly selected adult hospital inpatients records using the Global Trigger Tool | Median = 61 (45-74) | 12 months | NR | IHI Global Trigger | NR |
| Harugeri 2011 | 920 | India | Prospective | Hospital | Hospitalised medical Indian elderly inpatients of 60 years | Mean = 60.2 (18.2) | 2.5 years | Yes (ATC) | Shumock and Thornton scale | NR |
| Honhout 2010 | 7926 | Netherlands | Retrospective | Hospital | Patients admitted to 21 hospitals in 2004 | Median = 66 (60 to 108) | 12 months | Yes (ATC) | Other | Distinguishes between those discharge alive or died in hospital, also adverse reaction, prescription or dispensing |
| Howard 2003 | 4093 | US | Retrospective | Hospital | Patient records | 98% above 40 years | 6 months | Yes QSHC categories (most frequently reported) | Hepler criteria | Yes (prescribing monitoring, adherence |
| Hug 2010 | 1200 | US | Retrospective | Hospital | Patient records | Mean = 62.6 (20.7) | 18 months | Yes | Yes using CPOE | NR |
| Ithnin 2018 | 423 | Malaysia (Kuala Lumpur) | Prospective | Paediatric general ward and NICU | Two-pronged approach 1) Spontaneous reporting and 2) daily progress notes review | Mean = 74.6 (14.4) | 1 month | Yes (ATC) | Schumock and Thornton | NR |
| Jha 2001 | 3238 | US | Retrospective | Hospital | Patients admitted to medical and surgical unites | Adults | 8 months | Yes | No scale | NR |
| Jonsson 2010 | 1574 | Sweden | Retrospective | Hospital | Deceased patients from Fatal adverse drug reaction | Median = 86 (63-92) | 12 months | NR | Hallas K score | NR |
| Kaushal 2001 | 10778 | US | Prospective | Hospital | Paediatric, 6 weeks | Paediatric | 6 weeks | Yes (reports top 4) | Naranjo | Yes (ordering, transcribed, administrating, dispensing, monitoring) |
| Kaushal 2007 | 1788 | US | Prospective | Hospital | Ambulatory Paediatric patients under the age of 21 | Paediatric | 2 months | Yes | Physician assessment (no scale) | Yes (ordering, transcribed, administrating, dispensing, monitoring) |
| Klopotowska 2013 | 250 | Netherlands | Retrospective | Hospital | Older hospitalised patients | Mean = 76.9 (7.5) | 8 months | Yes ATC | IHI Trigger tool and CRF | Yes (prescribing administering monitoring) |
| Kopp 2006 | 185 | US | Prospective | Hospital | Patients in a medical/ surgical intensive care unit. | Mean = 53.2 (21.2) | 16.5 days | Yes | 4 point Likert scale | Yes |
| Kunac 2009 | 495 | New Zealand | Prospective | Pedriatic | Paediatric, incl. neonatal intensive care unit | Paediatric | 12 weeks | NR | novel standardised form | NR |
| Lagnaoui 2000 | 444 | France | Prospective | Internal Medicine | All patients admitted to a department of  internal medicine | Mean = 58.7 | 4 months | Yes | Preventability was assessed using an algorithm derived from a fatality preventability evaluation method | NR |
| Laroche 2013 | 1332 | France | Cross-sectional | General hospital | subjects were selected by random draw to be a representative sample of French patients with dementia | Mean = 82.0 ± 8.0 years (46–108) | 6 months | Yes | Yes | NR |
| Ligi 2008 | 388 | France | Prospective | Hospital | Neonatal intensive care unit | Neonates | 9 months | NR | 6 point Likert scale | Yes but not clear |
| López 2009 | 2582 | US | Prospective | Hospital | Adults above 18 , who were medical or surgical patients | More than 50% older than 50 years | 6 months | NR | own categories | sort of- event characteristics (newly prescribed drug, surgical procedures, tests and procedures) |
| Lovborg 2012 | 7322 | Sweden | Retrospective | Primary Care | All patient records indicating ADR | Median = 58 years old (range 0–99) | 2 years | Yes (ATC) | Hallas K score | Yes (prescribing, administration, monitoring) |
| Meier 2015 | 2262 | Germany | Prospective | Hospital | All adult non-trauma ED admissions | Mean = 62.1 (20.2) | 2 years | Yes (ATC) | Schumock and Thornton | NR |
| Miller 2006 | 8215 | Australia | Prospective | Primary Care | Patients visiting General Practice appointments | 45 years or over | 10 months | NR | No defined scale (GPs use own judgement) | NR |
| Morimoto 2011 | 3459 | Japan | Prospective | Hospital | Adults admitted to medical, surgical, or intensive care units in tertiary hospitals | Mean = 66 (17) | 6 months | Yes | No defined scale | Yes (ordering, transcription, dispensing, administration, monitoring) |
| Olivier 2002 | 671 | France | Prospective | Hospital | Patients over 15 admitted to an emergency department. | Mean = 55.6 (22.5) | 4 weeks | Yes ATC | Schumock and Thornton | NR |
| Park 2013 | 346 | South Korea | Retrospective | Intensive Care | Patients admitted to ICU over 4 months | Mean = 60.1 (14.6) | 4 months | Yes | Schumock and Thornton | NR |
| Patel 2007 | 6899 | India | Prospective | Emergency Department | Patients above the age of 18 | Mean = 40 | 6 weeks | Yes | Hallas K score | NR |
| Peyriere 2003 | 156 | France | Prospective | Internal Medicine | All patients admitted to the internal medicine | Mean = 66.5 | 6 x 8 days (48 days) | Yes (some) | No defined scale | NR |
| Phillips 2014 | 370 | Australia | Prospective | Hospital | Patients to hospital via emergency department | Mean = 65.36 (4.34) with ADE | 8 weeks | Yes (ATC) | Chan et al (avoid-ability) | NR |
| Pirmohamed 2004 | 18820 | UK | Prospective | Hospital | Patients admitted to 2 hospitals | Median = 76 (65-83) | 6 months | Yes | Hallas K score | NR |
| Pourseyed 2009 | 400 | Iran | Prospective | Internal Medicine | Patients admitted to the internal medicine ward. | Mean = 60.41 (13-91) | 15 weeks | No | Schumock and Thornton | NR |
| Rachana 2019 | 240 | India | Prospective | Hospital | Patients enrolled as per the study criteria then ADRs data was evaluated by assessment of causality, severity, and preventability. | Range (male): 4-83; Range (female): 1-68 | 8 months | Yes (ATC) | Schumock and Thornton | NR |
| Remesh 2014 | 550 | India | Prospective | Hospital | Patients who had experienced at least one suspected ADR after hospitalisation | Mean = 43.4 (18.5) | 6 months | Yes | Schumock and Thornton | NR |
| Rothschild 2007 | 1871 | US | Prospective | Psychiatry | Patients admitted to psychiatric hospital | Paediatric | 6 months | Yes | own scale | Yes (ordering, transcription, administration, other) |
| Sakuma 2014 | 1189 | Japan | Prospective | Hospital | Paediatric inpatients | Adults (over 18) | 3 months | sort of mentions the main ones not in a table | own scale | Yes (ordering, transcription, dispensing, administration, monitoring) |
| Schade 2006 | 1011 | US | Prospective | Hospitals | all adult inpatient discharges | Excluded new-borns | 6 month | Yes | No defined scale | NR |
| Senst 2001 | 3187 | US | Prospective | Internal Medicine | All admissions during  a 53-day study period | Above 60 years mostly suffered from ADRs | 2 months | Yes | No defined scale | Yes |
| Sriram 2011 | 3117 | India | Prospective | Internal Medicine | All patients of either sex and of any age who developed an ADR | Median = 3.9 (1.8-7.1) | 12 months | NR | Schumock and Thornton | NR |
| Sundaran 2018 | 3074 | India | Prospective | Hospital | ADRs reported by the health-care professionals, or the patients were confirmed with the physician-in-charge | Children | 7 months | Yes (Wills and Brown) | Schumock and Thornton | NR |
| Takata 2008 | 960 | US | Retrospective | Paediatric | Patients admitted to US children’s hospitals | Mean = 38.4 (22.6) | 8 weeks | Yes | no scale | Yes (monitoring, prescribing, dispensing, administration, transcribing) |
| Tangiisuran 2012 | 560 | UK | Prospective | Geriatric | Patients over 80 years of age admitted to elderly wards in hospitals | Paediatric | 2 x 3 month periods | Yes | Hallas K score | NR |
| Van der Hooft 2008 | 3515 | Netherlands | Prospective | Primary Care | Patients who had been registered with the GP | Mean = 49.6 (20.8) | 12 months | Yes (ATC) | Hallas K score | NR |
| Woo 2019 | 3202 | Canada | Retrospective | Emergency department | Patients who had been diagnosed with one or more medication-related problems or adverse drug events in one of three prospective multi-centre parent studies | Mean (sd) = 68.8 (18.0) | 12 months | NR | No defined scale | NR |
| Zandieh 2008 | 1689 | US | Prospective | Paediatric | Paediatrics patients under the age of 21 | NR, but 31-69 | 10 months | NR | no defined scale | NR |
| Zed 2008 | 1017 | US | Prospective | Emergency Department | ADR reports | Mean (sd) = 48.9 (20.4) | 12 weeks | Yes (see appendix) | no defined scale | NR |

NR: not reported; ADEs: adverse drug events; ATC: Anatomical Therapeutic Chemical (ATC) Classification System
