# Supplementary material for: Preventable medication harm across health care settings: a systematic review and meta-analysis
Source: BMC Med. 2020 Nov 6;18:313. doi: 10.1186/s12916-020-01774-9 (PMC7646069; doi:10.1186/s12916-020-01774-9)
Supplement: Supplementary file 5 — Additional file 5: Table S5. Critical appraisal ratings for all studies included in review. [file 12916_2020_1774_MOESM5_ESM.docx]

# **Additional file 5: Table S5: Critical appraisal ratings for all studies included in the review**

|  | Selection | | | | Comparability | Outcome | | | Preventability | | **Total score** |
| --- | --- | --- | --- | --- | --- | --- | --- | --- | --- | --- | --- |
| **Study** | 1) Representative of the exposed cohort | 2) Selection of the non-exposed cohort | 3) Ascertainment of exposure | 4) Outcome of interest not present at start of study | Comparability of cohorts on the basis of the design or analysis | 1) Assessment of outcome | 2) Was follow-up long enough for outcomes to occur | 3) Adequacy of follow up of cohorts | 1) Clear description of process to establish preventability | 2) Standardized methods to assess preventability |  |
| Ahern 2014 | 1 | 1 | 1 | 1 | 1 | 1 | 0 | 1 | 1 | 1 | **9** |
| Al Damen 2019 | 1 | 1 | 1 | 1 | 0 | 0 | 1 | 1 | 1 | 1 | **8** |
| Alam 2014 | 0 | 1 | 1 | 1 | 1 | 1 | 0 | 0 | 1 | 1 | **7** |
| Aljadhey 2013 | 1 | 1 | 0 | 1 | 1 | 1 | 0 | 0 | 0 | 0 | **5** |
| Alsbou 2010 | 1 | 1 | 1 | 0 | 0 | 1 | 0 | 0 | 1 | 1 | **6** |
| Alsbou 2015 | 1 | 1 | 1 | 1 | 1 | 1 | 1 | 1 | 1 | 1 | **10** |
| Al-Tajir 2005 | 1 | 1 | 0 | 1 | 0 | 0 | 1 | 1 | 1 | 1 | **7** |
| Ayani 2016 | 0 | 1 | 1 | 1 | 0 | 1 | 1 | 1 | 0 | 0 | **6** |
| Benkirane 2009a | 0 | 1 | 1 | 1 | 0 | 1 | 0 | 0 | 1 | 1 | **6** |
| Benkirane 2009b | 1 | 1 | 1 | 1 | 1 | 1 | 1 | 1 | 0 | 0 | **8** |
| Bernad-Laribiere 2015 | 0 | 1 | 1 | 1 | 1 | 1 | 0 | 0 | 1 | 0 | **6** |
| Buckley 2007 | 0 | 1 | 0 | 0 | 0 | 0 | 1 | 1 | 1 | 1 | **5** |
| Calderon-Ospina 2010 | 1 | 1 | 1 | 1 | 1 | 1 | 1 | 1 | 1 | 1 | **10** |
| Carayon 2014 | 1 | 1 | 1 | 0 | 1 | 1 | 1 | 1 | 0 | 0 | **7** |
| Castro 2013 | 1 | 1 | 0 | 1 | 1 | 1 | 1 | 1 | 1 | 0 | **8** |
| Chan 2001 | 0 | 1 | 1 | 1 | 1 | 1 | 0 | 0 | 1 | 1 | **7** |
| Chanie Eshetie 2015 | 1 | 1 | 1 | 1 | 1 | 1 | 1 | 1 | 1 | 1 | **10** |
| Chen 2012 | 1 | 1 | 1 | 1 | 1 | 1 | 0 | 0 | 1 | 1 | **8** |
| Damen 2017 | 1 | 1 | 1 | 1 | 1 | 1 | 1 | 1 | 1 | 0 | **9** |
| Davies 2009 | 1 | 1 | 1 | 1 | 1 | 1 | 1 | 1 | 1 | 1 | **10** |
| Davies 2010 | 1 | 1 | 1 | 1 | 1 | 1 | 1 | 1 | 1 | 1 | **10** |
| de Boer 2013 | 0 | 1 | 1 | 1 | 1 | 1 | 1 | 1 | 0 | 0 | **7** |
| Dequito 2011 | 1 | 1 | 1 | 1 | 1 | 1 | 1 | 1 | 1 | 0 | **9** |
| Easton 2003 | 1 | 1 | 0 | 1 | 0 | 0 | 0 | 0 | 1 | 1 | **5** |
| Farcas 2010 | 1 | 1 | 1 | 1 | 1 | 0 | 0 | 0 | 1 | 0 | **6** |
| Farcas 2014 | 1 | 1 | 1 | 1 | 1 | 1 | 1 | 1 | 1 | 1 | **10** |
| Forster 2004 | 1 | 1 | 1 | 1 | 0 | 1 | 0 | 0 | 1 | 1 | **7** |
| Forster 2005 | 1 | 1 | 1 | 1 | 1 | 1 | 1 | 1 | 1 | 1 | **10** |
| Franceschi 2008 | 0 | 1 | 1 | 1 | 1 | 1 | 1 | 1 | 1 | 1 | **9** |
| Gallagher 2012 | 0 | 1 | 1 | 1 | 1 | 1 | 1 | 1 | 1 | 1 | **9** |
| Gandhi 2003 | 1 | 1 | 1 | 1 | 1 | 1 | 1 | 1 | 1 | 1 | **10** |
| Geer 2016 | 1 | 1 | 1 | 1 | 1 | 1 | 1 | 1 | 1 | 1 | **10** |
| Grenouillet-Delacre 2007 | 1 | 1 | 1 | 1 | 1 | 1 | 1 | 1 | 1 | 1 | **10** |
| Gurwitz 2000 | 1 | 1 | 1 | 1 | 1 | 1 | 1 | 1 | 1 | 1 | **10** |
| Gurwitz 2003 | 1 | 1 | 1 | 1 | 0 | 1 | 1 | 1 | 1 | 1 | **9** |
| Gurwitz 2005 | 1 | 1 | 1 | 1 | 1 | 1 | 0 | 0 | 1 | 1 | **8** |
| Haile 2013 | 1 | 1 | 1 | 1 | 1 | 1 | 1 | 1 | 1 | 1 | **10** |
| Hamilton 2011 | 0 | 1 | 1 | 1 | 1 | 1 | 1 | 1 | 1 | 1 | **9** |
| Hardmeier 2004 | 1 | 1 | 1 | 0 | 0 | 1 | 1 | 1 | 0 | 0 | **6** |
| Harkanen 2015 | 1 | 1 | 1 | 1 | 1 | 1 | 0 | 0 | 1 | 1 | **8** |
| Harugeri 2011 | 0 | 1 | 1 | 1 | 1 | 1 | 1 | 1 | 1 | 1 | **9** |
| Honhout 2010 | 1 | 1 | 1 | 1 | 0 | 1 | 0 | 0 | 1 | 1 | **7** |
| Howard 2003 | 1 | 1 | 1 | 1 | 1 | 1 | 0 | 0 | 1 | 0 | **7** |
| Hug 2010 | 1 | 1 | 1 | 1 | 1 | 1 | 0 | 0 | 1 | 1 | **8** |
| Ithnin 2018 | 1 | 1 | 1 | 1 | 1 | 1 | 0 | 0 | 1 | 1 | **8** |
| Jha 2001 | 1 | 1 | 1 | 0 | 0 | 1 | 0 | 0 | 0 | 0 | **4** |
| Jonsson 2010 | 1 | 0 | 1 | 0 | 0 | 1 | 0 | 0 | 1 | 1 | **5** |
| Kaushal 2001 | 1 | 1 | 1 | 1 | 0 | 1 | 0 | 0 | 1 | 1 | **7** |
| Kaushal 2007 | 0 | 1 | 0 | 0 | 1 | 0 | 1 | 0 | 0 | 0 | **3** |
| Klopotowska 2013 | 1 | 1 | 1 | 1 | 0 | 1 | 0 | 0 | 1 | 1 | **7** |
| Kopp 2006 | 1 | 1 | 0 | 1 | 1 | 1 | 0 | 0 | 0 | 0 | **5** |
| Kunac 2009 | 1 | 1 | 1 | 1 | 0 | 1 | 0 | 0 | 1 | 0 | **6** |
| Lagnaoui 2000 | 1 | 1 | 1 | 1 | 1 | 1 | 1 | 1 | 0 | 0 | **8** |
| Laroche 2013 | 1 | 1 | 1 | 1 | 1 | 1 | 1 | 1 | 1 | 1 | **10** |
| Ligi 2008 | 1 | 1 | 0 | 0 | 1 | 1 | 0 | 0 | 1 | 0 | **5** |
| López 2009 | 1 | 1 | 0 | 0 | 1 | 0 | 1 | 0 | 0 | 0 | **4** |
| Lovborg 2012 | 0 | 0 | 0 | 0 | 0 | 0 | 0 | 0 | 1 | 1 | **2** |
| Meier 2015 | 1 | 1 | 1 | 1 | 0 | 1 | 0 | 0 | 1 | 1 | **7** |
| Miller 2006 | 1 | 1 | 1 | 1 | 1 | 1 | 1 | 0 | 0 | 0 | **7** |
| Morimoto 2011 | 1 | 1 | 1 | 0 | 1 | 1 | 0 | 0 | 0 | 0 | **5** |
| Olivier 2002 | 1 | 1 | 1 | 1 | 0 | 1 | 0 | 0 | 1 | 1 | **7** |
| Park 2013 | 0 | 1 | 1 | 1 | 1 | 1 | 1 | 1 | 1 | 1 | **9** |
| Patel 2007 | 1 | 1 | 0 | 1 | 0 | 1 | 0 | 0 | 1 | 1 | **6** |
| Peyriere 2003 | 1 | 1 | 1 | 1 | 0 | 1 | 0 | 0 | 0 | 0 | **5** |
| Phillips 2014 | 1 | 1 | 1 | 1 | 0 | 1 | 0 | 0 | 1 | 1 | **7** |
| Pirmohamed 2004 | 1 | 1 | 1 | 1 | 0 | 1 | 0 | 0 | 1 | 1 | **7** |
| Pourseyed 2009 | 1 | 1 | 1 | 1 | 1 | 1 | 1 | 1 | 1 | 1 | **10** |
| Rachana 2019 | 1 | 1 | 1 | 0 | 0 | 0 | 0 | 0 | 1 | 0 | **4** |
| Remesh 2014 | 0 | 1 | 0 | 0 | 0 | 0 | 0 | 0 | 1 | 1 | **3** |
| Rothschild 2007 | 0 | 1 | 1 | 1 | 0 | 1 | 0 | 0 | 0 | 0 | **4** |
| Sakuma 2014 | 1 | 1 | 1 | 1 | 0 | 1 | 0 | 0 | 0 | 0 | **5** |
| Schade 2006 | 1 | 1 | 1 | 1 | 0 | 1 | 1 | 1 | 0 | 0 | **7** |
| Senst 2001 | 1 | 1 | 1 | 1 | 1 | 1 | 0 | 0 | 0 | 0 | **6** |
| Sriram 2011 | 1 | 1 | 0 | 0 | 0 | 0 | 0 | 0 | 1 | 1 | **4** |
| Sundaran 2018 | 0 | 1 | 1 | 0 | 0 | 0 | 1 | 1 | 1 | 1 | **6** |
| Takata 2008 | 1 | 1 | 1 | 1 | 0 | 1 | 1 | 1 | 0 | 0 | **7** |
| Tangiisuran 2012 | 1 | 1 | 1 | 1 | 1 | 1 | 1 | 1 | 1 | 1 | **10** |
| Van der Hooft 2008 | 1 | 1 | 1 | 1 | 1 | 1 | 0 | 0 | 1 | 1 | **8** |
| Woo 2019 | 1 | 1 | 0 | 0 | 1 | 1 | 1 | 0 | 0 | 0 | **5** |
| Zandieh 2008 | 1 | 1 | 0 | 0 | 1 | 0 | 1 | 1 | 0 | 0 | **5** |
| Zed 2008 | 1 | 1 | 0 | 0 | 1 | 0 | 1 | 1 | 0 | 0 | **5** |
