# Supplementary material for: Preventable medication harm across health care settings: a systematic review and meta-analysis
Source: BMC Med. 2020 Nov 6;18:313. doi: 10.1186/s12916-020-01774-9 (PMC7646069; doi:10.1186/s12916-020-01774-9)
Supplement: Supplementary file 6 — Additional file 6: Table S6.. Top five most common preventable medication harms by ATC drug classification. % represent prevalence’s. [file 12916_2020_1774_MOESM6_ESM.docx]

# **Additional file 6: Table S6: Top five most common preventable medication harms by ATC drug class, % represent prevalence’s**
