# Supplementary material for: Preventable medication harm across health care settings: a systematic review and meta-analysis
Source: BMC Med. 2020 Nov 6;18:313. doi: 10.1186/s12916-020-01774-9 (PMC7646069; doi:10.1186/s12916-020-01774-9)
Supplement: Supplementary file 7 — Additional file 7: Figure S1. Funnel plot of preventable medication harm (log-transformed proportion). [file 12916_2020_1774_MOESM7_ESM.docx]

# **Additional file 7: Fig. S1 Funnel plot of preventable medication harm (log-transformed proportion)**
